# Supplementary material for: Biopsychosocial Factors for Chronicity in Individuals with Non-Specific Low Back Pain: An Umbrella Review
Source: Int J Environ Res Public Health. 2022 Aug 16;19(16):10145. doi: 10.3390/ijerph191610145 (PMC9408093; doi:10.3390/ijerph191610145)
Supplement: Supplementary file 1 [file ijerph-19-10145-s001.zip › ijerph-1842562-supplementary.pdf]

Supplementary Table S1: PRISMA checklist

Section and Topic	Item #	Checklist item	Location where item is reported
**TITLE**			
Title	1	Identify the report as a systematic review.	Title page
**ABSTRACT**			
Abstract	2	See the PRISMA 2020 for Abstracts checklist.	Line 18-35
**INTRODUCTION**			
Rationale	3	Describe the rationale for the review in the context of existing knowledge.	Line 38-66
Objectives	4	Provide an explicit statement of the objective(s) or question(s) the review addresses.	Line 67-69
**METHODS**			
Eligibility criteria	5	Specify the inclusion and exclusion criteria for the review and how studies were grouped for the syntheses.	Line 97-109<br>Line 121-124
Information sources	6	Specify all databases, registers, websites, organisations, reference lists and other sources searched or consulted to identify studies. Specify the date when each source was last searched or consulted.	Line 83-95
Search strategy	7	Present the full search strategies for all databases, registers and websites, including any filters and limits used.	Supplementary Table S2
Selection process	8	Specify the methods used to decide whether a study met the inclusion criteria of the review, including how many reviewers screened each record and each report retrieved, whether they worked independently, and if applicable, details of automation tools used in the process.	Line 71-75
Data collection process	9	Specify the methods used to collect data from reports, including how many reviewers collected data from each report, whether they worked independently, any processes for obtaining or confirming data from study investigators, and if applicable, details of automation tools used in the process.	Line 73-75
Data items	10a	List and define all outcomes for which data were sought. Specify whether all results that were compatible with each outcome domain in each study were sought (e.g. for all measures, time points, analyses), and if not, the methods used to decide which results to collect.	Line 115-121
	10b	List and define all other variables for which data were sought (e.g. participant and intervention characteristics, funding sources). Describe any assumptions made about any missing or unclear information.	Line 111-114
Study risk of bias assessment	11	Specify the methods used to assess risk of bias in the included studies, including details of the tool(s) used, how many reviewers assessed each study and whether they worked independently, and if applicable, details of automation tools used in the process.	Line 139-148<br>Line 74
Effect measures	12	Specify for each outcome the effect measure(s) (e.g. risk ratio, mean difference) used in the synthesis or presentation of results.	Line 154-155<br>Line 124-127
Synthesis methods	13a	Describe the processes used to decide which studies were eligible for each synthesis (e.g. tabulating the study intervention characteristics and comparing against the planned groups for each synthesis (item #5)).	Line 73-74
	13b	Describe any methods required to prepare the data for presentation or synthesis, such as handling of missing summary statistics, or data conversions.	Line 128-137
	13c	Describe any methods used to tabulate or visually display results of individual studies and syntheses.	Line 150-151
	13d	Describe any methods used to synthesize results and provide a rationale for the choice(s). If meta-analysis was performed, describe the model(s), method(s) to identify the presence and extent of statistical heterogeneity, and software package(s) used.	Line 163-169
	13e	Describe any methods used to explore possible causes of heterogeneity among study results (e.g. subgroup analysis, meta-regression).	Line 166-169
	13f	Describe any sensitivity analyses conducted to assess robustness of the synthesized results.	N/A
Reporting bias assessment	14	Describe any methods used to assess risk of bias due to missing results in a synthesis (arising from reporting biases).	N/A
Certainty assessment	15	Describe any methods used to assess certainty (or confidence) in the body of evidence for an outcome.	N/A
**RESULTS**			
Study selection	16a	Describe the results of the search and selection process, from the number of records identified in the search to the number of studies included in the review, ideally using a flow diagram.	Figure
	16b	Cite studies that might appear to meet the inclusion criteria, but which were excluded, and explain why they were excluded.	Line 175-176
Study characteristics	17	Cite each included study and present its characteristics.	Table 1

Section and Topic	Item #	Checklist item	Location where item is reported
Risk of bias in studies	18	Present assessments of risk of bias for each included study.	Supplementary Table 7
Results of individual studies	19	For all outcomes, present, for each study: (a) summary statistics for each group (where appropriate) and (b) an effect estimate and its precision (e.g. confidence/credible interval), ideally using structured tables or plots.	Table 2
Results of syntheses	20a	For each synthesis, briefly summarise the characteristics and risk of bias among contributing studies.	Line 233-285
	20b	Present results of all statistical syntheses conducted. If meta-analysis was done, present for each the summary estimate and its precision (e.g. confidence/credible interval) and measures of statistical heterogeneity. If comparing groups, describe the direction of the effect.	Table 3
	20c	Present results of all investigations of possible causes of heterogeneity among study results.	Table 2<br>Supplementary Table S9
	20d	Present results of all sensitivity analyses conducted to assess the robustness of the synthesized results.	N/A
Reporting biases	21	Present assessments of risk of bias due to missing results (arising from reporting biases) for each synthesis assessed.	N/A
Certainty of evidence	22	Present assessments of certainty (or confidence) in the body of evidence for each outcome assessed.	N/A
**DISCUSSION**			
Discussion	23a	Provide a general interpretation of the results in the context of other evidence.	Line 287-372
	23b	Discuss any limitations of the evidence included in the review.	Line 381-388
	23c	Discuss any limitations of the review processes used.	Line 388-394
	23d	Discuss implications of the results for practice, policy, and future research.	Line 395-413
**OTHER INFORMATION**			
Registration and protocol	24a	Provide registration information for the review, including register name and registration number, or state that the review was not registered.	Line 80
	24b	Indicate where the review protocol can be accessed, or state that a protocol was not prepared.	Line 79-80
	24c	Describe and explain any amendments to information provided at registration or in the protocol.	Line 384-388
Support	25	Describe sources of financial or non-financial support for the review, and the role of the funders or sponsors in the review.	Line 436
Competing interests	26	Declare any competing interests of review authors.	Line 439
Availability of data, code and other materials	27	Report which of the following are publicly available and where they can be found: template data collection forms; data extracted from included studies; data used for all analyses; analytic code; any other materials used in the review.	Line 424-430

Supplementary Table S2: Search strategy for PubMed

#1	Search **dorsalgia [Title/Abstract]**	92
#2	Search **"Back Pain"[Mesh]**	38358
#3	Search **backache [Title/Abstract]**	3667
#4	Search **"Low Back Pain"[Mesh]**	21412
#5	Search **"lumbar pain"[Title/Abstract]**	1476
#6	Search **coccyx [Title/Abstract]**	833
#7	Search **coccydynia [Title/Abstract]**	136
#8	Search **sciatica [Title/Abstract]**	4739
#9	Search **"Sciatic Neuropathy"[Mesh:NoExp]**	2008
#10	Search **spondylosis [Title/Abstract]**	3423
#11	Search **lumbago [Title/Abstract]**	1347
#12	Search **back disorder$[Title/Abstract]**	137
#13	Search **((((((((((dorsalgia[Title/Abstract]) OR "Back Pain"[Mesh]) OR backache[Title/Abstract]) OR "Low Back Pain"[Mesh]) OR "lumbar pain"[Title/Abstract]) OR coccyx[Title/Abstract]) OR coccydynia[Title/Abstract]) OR sciatica[Title/Abstract]) OR "Sciatic Neuropathy"[Mesh:NoExp]) OR spondylosis[Title/Abstract]) OR lumbago[Title/Abstract]) OR back disorder$[Title/Abstract]**	50764
#14	Search **"Cohort Studies"[Mesh:NoExp]**	257229
#15	Search **incidence [Text Word]**	835714
#16	Search **"Mortality"[Mesh:NoExp]**	43314
#17	Search **"Follow-Up Studies"[Mesh:NoExp]**	636101
#18	Search **prognos*[Text Word]**	847092
#19	Search **predict*[Text Word]**	1618941
#20	Search **course[Text Word]**	545336
#21	Search **"Survival Analysis"[Mesh:NoExp]**	133063
#22	Search **((((((("Cohort Studies"[Mesh:NoExp]) OR incidence[Text Word]) OR "Mortality"[Mesh:NoExp]) OR "Follow-Up Studies"[Mesh:NoExp]) OR prognos*[Text Word]) OR predict*[Text Word]) OR course[Text Word]) OR "Survival Analysis"[Mesh:NoExp]**	4016575
#23	Search **"Meta-Analysis" [Publication Type]**	112164
#24	Search **systematic review[Text Word]**	170908
#25	Search **("Meta-Analysis" [Publication Type]) OR systematic review[Text Word]**	230609
#26	Search **(((((((((((((dorsalgia[Title/Abstract]) OR "Back Pain"[Mesh]) OR backache[Title/Abstract]) OR "Low Back Pain"[Mesh]) OR "lumbar pain"[Title/Abstract]) OR coccyx[Title/Abstract]) OR coccydynia[Title/Abstract]) OR sciatica[Title/Abstract]) OR "Sciatic Neuropathy"[Mesh:NoExp]) OR spondylosis[Title/Abstract]) OR lumbago[Title/Abstract]) OR back disorder$[Title/Abstract])) AND (((((((("Cohort Studies"[Mesh:NoExp]) OR incidence[Text Word]) OR "Mortality"[Mesh:NoExp]) OR "Follow-Up Studies"[Mesh:NoExp]) OR prognos*[Text Word]) OR predict*[Text Word]) OR course[Text Word]) OR "Survival Analysis"[Mesh:NoExp])) AND (("Meta-Analysis" [Publication Type]) OR systematic review[Text Word])**	280
#27	Search **(((((((((((((dorsalgia[Title/Abstract]) OR "Back Pain"[Mesh]) OR backache[Title/Abstract]) OR "Low Back Pain"[Mesh]) OR "lumbar pain"[Title/Abstract]) OR coccyx[Title/Abstract]) OR coccydynia[Title/Abstract]) OR sciatica[Title/Abstract]) OR "Sciatic Neuropathy"[Mesh:NoExp]) OR spondylosis[Title/Abstract]) OR**	269

	**lumbago[Title/Abstract]) OR back disorder$[Title/Abstract])) AND ((((((("Cohort Studies"[Mesh:NoExp]) OR incidence[Text Word]) OR "Mortality"[Mesh:NoExp]) OR "Follow-Up Studies"[Mesh:NoExp]) OR prognos*[Text Word]) OR predict*[Text Word]) OR course[Text Word]) OR "Survival Analysis"[Mesh:NoExp])) AND (("Meta-Analysis" [Publication Type]) OR systematic review[Text Word])** Filters: **Humans**	
#28	Search **((((((((((((dorsalgia[Title/Abstract]) OR "Back Pain"[Mesh]) OR backache[Title/Abstract]) OR "Low Back Pain"[Mesh]) OR "lumbar pain"[Title/Abstract]) OR coccyx[Title/Abstract]) OR coccydynia[Title/Abstract]) OR sciatica[Title/Abstract]) OR "Sciatic Neuropathy"[Mesh:NoExp]) OR spondylosis[Title/Abstract]) OR lumbago[Title/Abstract]) OR back disorder$[Title/Abstract])) AND ((((((("Cohort Studies"[Mesh:NoExp]) OR incidence[Text Word]) OR "Mortality"[Mesh:NoExp]) OR "Follow-Up Studies"[Mesh:NoExp]) OR prognos*[Text Word]) OR predict*[Text Word]) OR course[Text Word]) OR "Survival Analysis"[Mesh:NoExp])) AND (("Meta-Analysis" [Publication Type]) OR systematic review[Text Word])** Sort by: **Author** Filters: **Publication date from 2008/01/01 to 2020/03/20; Humans**	208

Supplementary Table S3: Reference from excluded full-text citations

1.	Alhowimel, A.; AlOtaibi, M.; Radford, K.; Coulson, N. Psychosocial Factors Associated with Change in Pain and Disability Outcomes in Chronic Low Back Pain Patients Treated by Physiotherapist: A Systematic Review. *SAGE open Med.* **2018**, *6*, doi:10.1177/2050312118757387.
2.	Alzahrani, H.; Mackey, M.; Stamatakis, E.; Zadro, J.R.; Shirley, D. The Association between Physical Activity and Low Back Pain: A Systematic Review and Meta-Analysis of Observational Studies. *Sci. Rep.* **2019**, *9*, 1–10, doi:10.1038/s41598-019-44664-8.
3.	Baumeister, H.; Knecht, A.; Hutter, N. Direct and Indirect Costs in Persons with Chronic Back Pain and Comorbid Mental Disorders--a Systematic Review. *J. Psychosom. Res.* **2012**, *73*, 79–85, doi:10.1016/j.jpsychores.2012.05.008.
4.	Buruck, G.; Tomaschek, A.; Wendsche, J.; Ochsmann, E.; Dörfel, D. Psychosocial Areas of Worklife and Chronic Low Back Pain: A Systematic Review and Meta-Analysis. *BMC Musculoskelet. Disord.* **2019**, *20*, 1–16, doi:10.1186/s12891-019-2826-3.
5.	da Costa, B.R.; Vieira, E.R. Risk Factors for Work-Related Musculoskeletal Disorders: A Systematic Review of Recent Longitudinal Studies. *Am. J. Ind. Med.* **2010**, *53*, 285–323, doi:10.1002/ajim.20750.
6.	Ferreira, P.H.; Pinheiro, M.B.; Machado, G.C.; Ferreira, M.L. Is Alcohol Intake Associated with Low Back Pain? A Systematic Review of Observational Studies. *Man. Ther.* **2013**, *18*, 183–190, doi:10.1016/j.math.2012.10.007.
7.	Konstantinou, K.; Hider, S.L.; Jordan, J.L.; Lewis, M.; Dunn, K.M.; Hay, E.M. The Impact of Low Back-Related Leg Pain on Outcomes as Compared With Low Back Pain Alone. *Clin. J. Pain* **2013**, *29*, 644–654, doi:10.1097/ajp.0b013e31826f9a52.
8.	Kuhnow, A.; Kuhnow, J.; Ham, D.; Rosedale, R. The McKenzie Method and Its Association with Psychosocial Outcomes in Low Back Pain: A Systematic Review. *Physiother. Theory Pract.* **2020**, *00*, 1–15, doi:10.1080/09593985.2019.1710881.
9.	Lee, H. Understanding Causal Mechanisms in Back Pain, Prince of Wales Clinical School Faculty of Medicine, 2017.
10.	Lee, H.; Hübscher, M.; Moseley, G.L.; Kamper, S.J.; Traeger, A.C.; Mansell, G.; McAuley, J.H. How Does Pain Lead to Disability? A Systematic Review and Meta-Analysis of Mediation Studies in People with Back and Neck Pain. *Pain* **2015**, *156*, 988–997, doi:10.1097/j.pain.0000000000000146.
11.	Nelson, N.A.; Hughes, R.E. Quantifying Relationships between Selected Work-Related Risk Factors and Back Pain: A Systematic Review of Objective Biomechanical Measures and Cost-Related Health Outcomes. *Int. J. Ind. Ergon.* **2009**, *39*, 202–210, doi:10.1016/j.ergon.2008.06.003.
12.	Parreira, P.; Maher, C.G.; Steffens, D.; Hancock, M.J.; Ferreira, M.L. Risk Factors for Low Back Pain and Sciatica: An Umbrella Review. *Spine J.* **2018**, *18*, 1715–1721, doi:10.1016/j.spinee.2018.05.018.
13.	Ranger, T.A.; Cicuttini, F.M.; Jensen, T.S.; Peiris, W.L.; Hussain, S.M.; Fairley, J.; Urquhart, D.M. Are the Size and Composition of the Paraspinal Muscles Associated with Low Back Pain? A Systematic Review. *Spine J.* **2017**, *17*, 1729–1748, doi:10.1016/j.spinee.2017.07.002.
14.	Saragiotto, B.T.; de Almeida, M.O.; Yamato, T.P.; Maher, C.G. Multidisciplinary Biopsychosocial Rehabilitation for Nonspecific Chronic Low Back Pain. *Phys. Ther.* **2016**, *96*, 759–763, doi:10.2522/ptj.20150359.
15.	Shiri, R.; Karppinen, J.; Leino-Arjas, P.; Solovieva, S.; Viikari-Juntura, E. The Association between Smoking and Low Back Pain: A Meta-Analysis. *Am. J. Med.* **2010**, *123*, 87.e7-35, doi:10.1016/j.amjmed.2009.05.028.
16.	Shiri, R.; Karppinen, J.; Leino-Arjas, P.; Solovieva, S.; Viikari-Juntura, E. The Association between Obesity and Low Back Pain: A Meta-Analysis. *Am. J. Epidemiol.* **2010**, *171*, 135–154, doi:10.1093/aje/kwp356.
17.	van den Berg, R.; Jongbloed, E.M.; de Schepper, E.I.T.; Bierma-Zeinstra, S.M.; Koes, B.W.; Luijsterburg, P.A.J. The Association between Pro-Inflammatory Biomarkers and Nonspecific Low Back Pain: A Systematic Review. *Spine J.* **2018**, *18*, 2140–2151, doi:10.1016/j.spinee.2018.06.349.
18.	Van Der Hulst, M.; Vollenbroek-Hutten, M.M.; Groothuis-Oudshoorn, K.G.; Hermens, H.J. Multidisciplinary Rehabilitation Treatment of Patients with Chronic Low Back Pain: A Prognostic Model for Its Outcome. *Clin. J. Pain* **2008**, *24*, 421–430, doi:10.1097/AJP.0b013e31816719f5.
19.	Cornelius, L.R.; van der Klink, J.J.; Groothoff, J.W.; Brouwer, S. Prognostic Factors of Long Term Disability Due to Mental Disorders: A Systematic Review. *J. Occup. Rehabil.* **2011**, *21*, 259–274, doi:10.1007/s10926-010-9261-5.
20.	Dekkers-Sanchez, P.M.; Hoving, J.L.; Sluiter, J.K.; Frings-Dresen, M.H. Factors Associated with Long-Term Sick Leave in Sick-Listed Employees: A Systematic Review. *Occup. Environ. Med.* **2008**, *65*, 153–157, doi:10.1136/oem.2007.034983.
21.	Jackson, T.; Wang, Y.; Wang, Y.; Fan, H. Self-Efficacy and Chronic Pain Outcomes: A Meta-Analytic Review. *J. Pain* **2014**, *15*, 800–814, doi:10.1016/j.jpain.2014.05.002.
22.	Martinez-Calderon, J.; Flores-Cortes, M.; Morales-Asencio, J.M.; Luque-Suarez, A. Pain-Related Fear, Pain Intensity and Function in Individuals With Chronic Musculoskeletal Pain: A Systematic Review and Meta-Analysis. *J. Pain* **2019**, *20*, 1394–1415, doi:10.1016/j.jpain.2019.04.009.
23.	Meints, S.M.; Edwards, R.R. Evaluating Psychosocial Contributions to Chronic Pain Outcomes. *Prog. Neuro-Psychopharmacology Biol. Psychiatry* **2018**, *87*, 168–182, doi:10.1016/j.pnpbp.2018.01.017.
24.	Ojha, H.A.; Wyrsta, N.J.; Davenport, T.E.; Egan, W.E.; Gellhorn, A.C. Timing of Physical Therapy Initiation for Nonsurgical Management of Musculoskeletal Disorders and Effects on Patient Outcomes: A Systematic Review. *J. Orthop. Sports Phys. Ther.* **2016**, *46*, 56–70, doi:10.2519/jospt.2016.6138.
25.	Peerdeman, K.J.; van Laarhoven, A.I.; Peters, M.L.; Evers, A.W. An Integrative Review of the Influence of Expectancies on Pain. *Front. Psychol.* 2016, *7*, 1270.
26.	Snippen, N.C.; de Vries, H.J.; van der Burg-Vermeulen, S.J.; Hagedoorn, M.; Brouwer, S. Influence of Significant Others on Work Participation of Individuals with Chronic Diseases: A Systematic Review. *BMJ Open* **2019**, *9*, e021742, doi:10.1136/bmjopen-2018-021742.
27.	Hruschak, V.; Cochran, G. Psychosocial Predictors in the Transition from Acute to Chronic Pain: A Systematic Review. *Psychol. Heal. Med.* **2018**, *23*, 1151–1167, doi:10.1080/13548506.2018.1446097.
28.	Shiri, R.; Falah-Hassani, K. Does Leisure Time Physical Activity Protect against Low Back Pain? Systematic Review and Meta-Analysis of 36 Prospective Cohort Studies. *Br. J. Sports Med.* **2017**, *51*, 1410–1418, doi:10.1136/bjsports-2016-097352.
29.	Artus, M.; Campbell, P.; Mallen, C.D.; Dunn, K.M.; van der Windt, D.A. Generic Prognostic Factors for Musculoskeletal Pain in Primary Care: A Systematic Review. *BMJ Open* **2017**, *7*, e012901, doi:10.1136/bmjopen-2016-012901.
30.	Buscemi, V.; Chang, W.J.; Liston, M.B.; McAuley, J.H.; Schabrun, S.M. The Role of Perceived Stress and Life Stressors in the Development of Chronic Musculoskeletal Pain Disorders: A Systematic Review. *J. Pain* **2019**, *20*, 1127–1139, doi:10.1016/j.jpain.2019.02.008.
31.	Campbell, P.; Wynne-Jones, G.; Dunn, K.M. The Influence of Informal Social Support on Risk and Prognosis in Spinal Pain: A Systematic Review. *Eur. J. Pain* **2011**, *15*, 444.e1-14, doi:10.1016/j.ejpain.2010.09.011.
32.	De Raaij, E.J.; Ostelo, R.W.; Maissan, F.; Mollema, J.; Wittink, H. The Association of Illness Perception and Prognosis for Pain and Physical Function in Patients With Noncancer Musculoskeletal Pain: A Systematic Literature Review. *J. Orthop. Sport. Phys. Ther.* **2018**, *48*, 789–800, doi:10.2519/jospt.2018.8072.
33.	Driessen, M.T.; Proper, K.I.; van Tulder, M.W.; Anema, J.R.; Bongers, P.M.; van der Beek, A.J.; AJ, D.M.P.K. van T.M.A.J.B.P. van der B. The Effectiveness of Physical and Organisational Ergonomic Interventions on Low Back Pain and Neck Pain: A Systematic Review [with Consumer Summary]. *Occup. Environ. Med.* **2010**, *67*, 277–285, doi:10.1136/oem.2009.047548.
34.	Ebrahim, S.; Malachowski, C.; Kamal El Din, M.; Mulla, S.M.; Montoya, L.; Bance, S.; Busse, J.W. Measures of Patients' Expectations about Recovery: A Systematic Review. *J. Occup. Rehabil.* **2015**, *25*, 240–255, doi:10.1007/s10926-014-9535-4.
35.	Kuijer, P.P.; Gouttebarge, V.; Brouwer, S.; Reneman, M.F.; Frings-Dresen, M.H. Are Performance-Based Measures Predictive of Work Participation in Patients with Musculoskeletal Disorders? A Systematic Review. *Int. Arch. Occup. Environ. Health* **2012**, *85*, 109–123, doi:10.1007/s00420-011-0659-y.
36.	Laisné, F.; Lecomte, C.; Corbière, M. Biopsychosocial Predictors of Prognosis in Musculoskeletal Disorders: A Systematic Review of the Literature (Corrected and Republished) *. *Disabil. Rehabil.* **2012**, *34*, 1912–1941, doi:10.3109/09638288.2012.729362.
37.	Luque-Suarez, A.; Martinez-Calderon, J.; Falla, D. Role of Kinesiophobia on Pain, Disability and Quality of Life in People Suffering from Chronic Musculoskeletal Pain: A Systematic Review. *Br. J. Sports Med.* **2019**, *53*, 554–559, doi:10.1136/bjsports-2017-098673.
38.	Martinez-Calderon, J.; Jensen, M.P.; Morales-Asencio, J.M.; Luque-Suarez, A. Pain Catastrophizing and Function in Individuals with Chronic Musculoskeletal Pain. *Clin. J. Pain* **2019**, *35*, 279–293, doi:10.1097/AJP.0000000000000676.
39.	Martinez-Calderon, J.; Zamora-Campos, C.; Navarro-Ledesma, S.; Luque-Suarez, A. The Role of Self-Efficacy on the Prognosis of Chronic Musculoskeletal Pain: A Systematic Review. *J. Pain* **2018**, *19*, 10–34, doi:10.1016/j.jpain.2017.08.008.
40.	Prang, K.-H.; Newnam, S.; Berecki-Gisolf, J. The Impact of Family and Work-Related Social Support on Musculoskeletal Injury Outcomes: A Systematic Review. *J. Occup. Rehabil.* **2015**, *25*, 207–219, doi:10.1007/s10926-014-9523-8.

41.	Runge, N.; Aina, A.; May, S. Are within and/or between Session Improvements in Pain and Function Prognostic of Medium and Long-Term Improvements in Musculoskeletal Problems? A Systematic Review. *Musculoskelet. Sci. Pract.* **2020**, *45*, 102102, doi:10.1016/j.msksp.2019.102102.
42.	Tseli, E.; Boersma, K.; Stålnacke, B.M.; Enthoven, P.; Gerdle, B.; Äng, B.O.; Grooten, W.J.A. Prognostic Factors for Physical Functioning After Multidisciplinary Rehabilitation in Patients With Chronic Musculoskeletal Pain: A Systematic Review and Meta-Analysis. *Clin. J. Pain* **2019**, *35*, 148–173, doi:10.1097/AJP.0000000000000669.
43.	Valentin, G.H.; Pilegaard, M.S.; Vaegter, H.B.; Rosendal, M.; Ørtenblad, L.; Væggemose, U.; Christensen, R. Prognostic Factors for Disability and Sick Leave in Patients with Subacute Non-Malignant Pain: A Systematic Review of Cohort Studies. *BMJ Open* **2016**, *6*, e007616, doi:10.1136/bmjopen-2015-007616.
44.	Menezes Costa, L. da C.; Maher, C.G.; Hancock, M.J.; McAuley, J.H.; Herbert, R.D.; Costa, L.O.P. The Prognosis of Acute and Persistent Low-Back Pain: A Meta-Analysis. *Can. Med. Assoc. J.* **2012**, *184*, E613-24, doi:10.1503/cmaj.111271.
45.	Rashid, M.; Kristofferzon, M.-L.; Nilsson, A.; Heiden, M. Factors Associated with Return to Work among People on Work Absence Due to Long-Term Neck or Back Pain: A Narrative Systematic Review. *BMJ Open* **2017**, *7*, e014939, doi:10.1136/bmjopen-2016-014939.
46.	Scheele, J.; Luijsterburg, P.A.; Bierma-Zeinstra, S.M.; Koes, B.W. Course of Back Complaints in Older Adults: A Systematic Literature Review. *Eur. J. Phys. Rehabil. Med.* **2012**, *48*, 379–386.
47.	Suri, P.; Fry, A.L.; Gellhorn, A.C. Do Muscle Characteristics on Lumbar Spine Magnetic Resonance Imaging or Computed Tomography Predict Future Low Back Pain, Physical Function, or Performance? A Systematic Review. *PM R* **2015**, *7*, 1269–1281, doi:10.1016/j.pmrj.2015.04.016.
48.	Iles, R.A.; Davidson, M.; Taylor, N.F. Psychosocial Predictors of Failure to Return to Work in Non-Chronic Non-Specific Low Back Pain: A Systematic Review. *Occup. Environ. Med.* **2008**, *65*, 507–517, doi:10.1136/oem.2007.036046.
49.	Ramond, A.; Bouton, C.; Richard, I.; Roquelaure, Y.; Baufreton, C.; Legrand, E.; Huez, J.-F. Psychosocial Risk Factors for Chronic Low Back Pain in Primary Care-a Systematic Review. *Fam. Pract.* **2011**, *28*, 12–21, doi:10.1093/fampra/cmq072.
50.	Heitz, C.A.; Hilfiker, R.; Bachmann, L.M.; Joronen, H.; Lorenz, T.; Uebelhart, D.; Klipstein, A.; Brunner, F. Comparison of Risk Factors Predicting Return to Work between Patients with Subacute and Chronic Non-Specific Low Back Pain: Systematic Review. *Eur Spine J* **2009**, *18*, 1829–1835, doi:10.1007/s00586-009-1083-9.
51.	Bartys, S.; Frederiksen, P.; Bendix, T.; Burton, K. System Influences on Work Disability Due to Low Back Pain: An International Evidence Synthesis. *Health Policy (New. York).* **2017**, *121*, 903–912, doi:10.1016/j.healthpol.2017.05.011.
52.	Melloh, M.; Elfering, A.; Egli Presland, C.; Roeder, C.; Barz, T.; Rolli Salathé, C.; Tamcan, O.; Mueller, U.; Theis, J.C. Identification of Prognostic Factors for Chronicity in Patients with Low Back Pain: A Review of Screening Instruments. *Int. Orthop.* **2009**, *33*, 301–313, doi:10.1007/s00264-008-0707-8.
53.	Verkerk, K.; Luijsterburg, P.A.; Miedema, H.S.; Pool-Goudzwaard, A.; Koes, B.W. Prognostic Factors for Recovery in Chronic Nonspecific Low Back Pain: A Systematic Review. *Phys. Ther.* **2012**, *92*, 1093–1108, doi:10.2522/ptj.20110388.
54.	Steenstra, I.A.; Munhall, C.; Irvin, E.; Oranye, N.; Passmore, S.; Van Eerd, D.; Mahood, Q.; Hogg-Johnson, S. Systematic Review of Prognostic Factors for Return to Work in Workers with Sub Acute and Chronic Low Back Pain. *J. Occup. Rehabil.* **2017**, *27*, 369–381, doi:10.1007/s10926-016-9666-x.
55.	Buchbinder, R.; Underwood, M. Prognosis in People with Back Pain. *Cmaj* **2012**, *184*, 1229–1230, doi:10.1503 /cmaj.111271.
56.	Arnold, E.; La Barrie, J.; DaSilva, L.; Patti, M.; Goode, A.; Clewley, D. The Effect of Timing of Physical Therapy for Acute Low Back Pain on Health Services Utilization: A Systematic Review. *Arch. Phys. Med. Rehabil.* **2019**, *100*, 1324–1338, doi:10.1016/j.apmr.2018.11.025.

Supplementary Table S4: Conflicts of interest of included studies

**First Author, year**	**Conflict of interest**
Kent PM, 2008	Not reported
Chou R, 2010	Financial Disclosures: None reported.
Steenstra IA, 2011	This new systematic review, updated from the previous one published in 2005, was funded by the Workers Compensation Board of Manitoba (WCB) and undertaken by a research team at the Institute for Work & Health.
Agnello A, 2010	No conflict of interest
Campbell P, 2013	No conflict of interest
Iles RA, 2009	Mr. Ross Iles is supported by an Australian Postgraduate Award and is a participant in the Work Disability Prevention CIHR Strategic Training Programme at the Universite de Sherbrooke, Canada.
Hallegraeff JM, 2012	Not reported
Hayden JA, 2019	No conflict of interest
Wertli & Rasmussen-Barr, 2014	No conflict of interest
Wertli & Eugster, 2014	No conflict of interest
Wertli & Burgstaller, 2014	No conflict of interest
Pinheiro MB, 2016	This research received no specific grant from any funding agency in the public, commercial, or not-for-profit sectors. MBP holds the International Post-graduate Research Scholarship/Post-graduate Award from the Australian Government. CGM's research fellowship is funded by Australia's National Health and Medical Research Council. MLF holds a Sydney Medical Foundation Fellowship, The University of Sydney.
Hendrick P, 2011	Not reported
Oliveira CB, 2019	Not reported
Wong AY, 2013	No conflict of interest

Supplementary Table S5: Primary studies (referenced in tables)

1.	Gatchel, R.J.; Polatin, P.B.; Mayer, T.G. The Dominant Role of Psychosocial Risk Factors in the Development of Chronic Low Back Pain Disability. *Spine (Phila. Pa. 1976).* **1995**, *20*, 2702–9, doi:10.1097/00007632-199512150-00011.
2.	Dixon, A.N.; Gatchel, R.J. Gender and Parental Status as Predictors of Chronic Low Back Pain Disability: A Prospective Study. *J. Occup. Rehabil.* **1999**, *9*, 195–200, doi:10.1023/A:1021353818130.
3.	Bakker, E.W.P.; Verhagen, A.P.; Lucas, C.; Koning, H.J.C.M.F.; Koes, B.W. Spinal Mechanical Load: A Predictor of Persistent Low Back Pain? A Prospective Cohort Study. *Eur. Spine J.* **2007**, *16*, 933–941, doi:10.1007/s00586-007-0347-5.
4.	Grotle, M.; Brox, J.I.; Glomsrød, B.; Lønn, J.H.; Vøllestad, N.K. Prognostic Factors in First-Time Care Seekers Due to Acute Low Back Pain. *Eur. J. Pain* **2007**, *11*, 290–298, doi:10.1016/j.ejpain.2006.03.004.
5.	Turner, J.A.; Franklin, G.; Fulton-Kehoe, D.; Sheppard, L.; Wickizer, T.M.; Wu, R.; Gluck, J. V.; K., E. Worker Recovery Expectations and Fear-Avoidance Predict Work Disability in a Population-Based Workers' Compensation Back Pain Sample. *Spine (Phila. Pa. 1976).* **2006**, *31*, 682–689, doi:10.1097/01.brs.0000202762.88787.af.
6.	Werneke, M.W.; Hart, D.L. Categorizing Patients With Occupational Low Back Pain by Use of the Quebec Task Force Classification System Versus Pain Pattern Classification Procedures: Discriminant and Predictive Validity. *Phys. Ther.* **2004**, *84*, 243–254, doi:10.1093/ptj/84.3.243.
7.	Cherkin, D.C.; Deyo, R.A.; Street, J.H.; Barlow, W. Predicting Poor Outcomes for Back Pain Seen in Primary Care Using Patients' Own Criteria. *Spine (Phila. Pa. 1976).* **1996**, *21*, 2900–2907, doi:10.1097/00007632-199612150-00023.
8.	Oleske, D.M.; Andersson, G.B.; Lavender, S.A.; Hahn, J.J. Association between Recovery Outcomes for Work-Related Low Back Disorders and Personal, Family, and Work Factors. *Spine (Phila. Pa. 1976).* **2000**, *25*, 1259–1265, doi:10.1097/00007632-200005150-00010.
9.	Schiøttz-Christensen, B.; Nielsen, G.L.; Hansen, V.K.; Schødt, T.; Sørensen, H.T.; Olesen, F. Long-Term Prognosis of Acute Low Back Pain in Patients Seen in General Practice: A 1-Year Prospective Follow-up Study. *Fam. Pract.* **1999**, *16*, 223–232, doi:10.1093/fampra/16.3.223.
10.	Thomas, E.; Silman, A.J.; Croft, P.R.; Papageorgiou, A.C.; Jayson, M.I. V. Predicting Who Develops Chronic Low Back Pain in Primary Care: A Prospective Study. *Gen. Pract.* **1999**, *318*, 1662–1667, doi:10.1136/bmj.318.7199.1662.
11.	Fransen, M.; Woodward, M.; Norton, R.; Coggan, C.; Dawe, M.; Sheridan, N. *Risk Factors Associated With the Transition From Acute to Chronic Occupational Back Pain*; 2002; Vol. 27;.
12.	Turner, J.A.; Franklin, G.; Fulton-Kehoe, D.; Sheppard, L.; Stover, B.; Wu, R.; Gluck, J. V.; Wickizer, T.M. ISSLS Prize Winner: Early Predictors of Chronic Work Disability. *Spine (Phila. Pa. 1976).* **2008**, *33*, 2809–2818, doi:10.1097/brs.0b013e31817df7a7.
13.	Haldorsern, E.M.; Kronholmb, K.; Skouenc, J.S.; Ursina, H. *Predictors for Outcome of a Multi-Modal Cognitive Behavioural Treatment Program for Low Back Pain Patients-a 12-Month Follow-up Study*; 1998; Vol. 2;.
14.	Oleinick, A.; Gluck, J. V.; Guire, K.E. Factors Affecting First Return to Work Following a Compensable Occupational Back Injury. *Am. J. Ind. Med.* **1996**, *30*, 540–555, doi:10.1002/(SICI)1097-0274(199611)30:5<540::AID-AJIM3>3.0.CO;2-X.
15.	Van Der Weide, W.E.; Verbeek, J.H.A.M.; Sallé, H.J.A.; Van Dijk, F.J.H. Prognostic Factors for Chronic Disability from Acute Low-Back Pain in Occupational Health Care. *Scand. J. Work. Environ. Heal.* **1999**, *25*, 50–56, doi:10.5271/sjweh.383.
16.	Du Bois, M.; Donceel, P. A Screening Questionnaire to Predict No Return to Work within 3 Months for Low Back Pain Claimants. *Eur. Spine J.* **2008**, *17*, 380–385, doi:10.1007/s00586-007-0567-8.
17.	van Doorn, J.W. Low Back Disability among Self-Employed Dentists, Veterinarians, Physicians and Physical Therapists in The Netherlands. A Retrospective Study over a 13-Year Period (N = 1,119) and an Early Intervention Program with 1-Year Follow-up (N = 134). *Acta Orthop. Scand.* **1995**, 1–64.
18.	Abenhaim, L.; Rossignol, M.; Gobeille, D.; Bonvalot, Y.; Fines, P.; Scott, S. The Prognostic Consequences in the Making of the Initial Medical Diagnosis of Work-Related Back Injuries. *Spine (Phila. Pa. 1976).* **1995**, *20*, 791–795, doi:https://doi.org/10.1097/00007632-199504000-00010.
19.	Seferlis, T.; Németh, G.; Carlsson, A.M. Prediction of Functional Disability, Recurrences, and Chronicity after 1 Year in 180 Patients Who Required Sick Leave for Acute Low-Back Pain. *J. Spinal Disord.* **2000**, *13*, 470–477, doi:10.1097/00002517-200012000-00002.
20.	Du Bois, M.; Szpalski, M.; Donceel, P. Patients at Risk for Long-Term Sick Leave Because of Low Back Pain. *Spine J.* **2009**, *9*, 350–359, doi:10.1016/j.spinee.2008.07.003.
21.	Nordin, M.; Skovron, M.L.; Hiebert, R.; Weiser, S.; Brisson, P.M.; Campello, M.; Crane, M.; Lewis, S. Early Predictors of Outcome. *Bull. Hosp. Jt. Dis.* **1996**, *55*, 204–206.
22.	Wahlgren, D.R.; Atkinson, J.H.; Epping-jordan, J.E.; Grant, I.; Webster, J.S.; Slater, M.A. One-Year Follow-up of First Onset Low Back Pain. *Pain* **1997**, *73*, 213–221, doi:https://doi.org/10.1016/s0304-3959(97)00106-1.
23.	Melloh, M.; Elfering, A.; Stanton, T.R.; Käser, A.; Salathé, C.R.; Barz, T.; Röder, C.; Theis, J.C. Who Is Likely to Develop Persistent Low Back Pain? A Longitudinal Analysis of Prognostic Occupational Factors. *Work* **2013**, *46*, 297–311, doi:10.3233/WOR-131672.
24.	Grotle, M.; Foster, N.E.; Dunn, K.M.; Croft, P. Are Prognostic Indicators for Poor Outcome Different for Acute and Chronic Low Back Pain Consulters in Primary Care? *Pain* **2010**, *151*, 790–797, doi:10.1016/j.pain.2010.09.014.
25.	Melloh, M.; Elfering, A.; Chapple, C.M.; Käser, A.; Salathé, C.R.; Barz, T.; Röder, C.; Theis, J.C. Prognostic Occupational Factors for Persistent Low Back Pain in Primary Care. *Int. Arch. Occup. Environ. Health* **2013**, *86*, 261–269, doi:10.1007/s00420-012-0761-9.
26.	Hiebert, R.; Campello, M.A.; Weiser, S.; Ziemke, G.W.; Fox, B.A.; Nordin, M. Predictors of Short-Term Work-Related Disability among Active Duty US Navy Personnel: A Cohort Study in Patients with Acute and Subacute Low Back Pain. *Spine J.* **2012**, *12*, 806–816, doi:10.1016/j.spinee.2011.11.012.
27.	Melloh, M.; Elfering, A.; Salathé, C.R.; Käser, A.; Barz, T.; Röder, C.; Theis, J.-C. Predictors of Sickness Absence in Patients with a New Episode of Low Back Pain in Primary Care. *Ind. Health* **2012**, *50*, 288–298, doi:10.2486/indhealth.MS1335.
28.	Reme, S.E.; Shaw, W.S.; Steenstra, I.A.; Woiszwillo, M.J.; Pransky, G.; Linton, S.J. Distressed, Immobilized, or Lacking Employer Support? A Sub-Classification of Acute Work-Related Low Back Pain. *J. Occup. Rehabil.* **2012**, *22*, 541–552, doi:10.1007/s10926-012-9370-4.
29.	Scheele, J.; Enthoven, W.T.M.; Bierma-Zeinstra, S.M.A.; Peul, W.C.; Van Tulder, M.W.; Bohnen, A.M.; Berger, M.Y.; Koes, B.W.; Luijsterburg, P.A.J. Course and Prognosis of Older Back Pain Patients in General Practice: A Prospective Cohort Study. *Pain* **2013**, *154*, 951–957, doi:10.1016/j.pain.2013.03.007.
30.	Valat, J.P.; Goupille, P.; Védere, V. Low Back Pain: Risk Factors for Chronicity. *Rev Rhum Engl Ed* **1997**, *64*, 189–197.
31.	Grotle, M.; Vøllestad, N.K.; Brox, J.I. Clinical Course and Impact of Fear-Avoidance Beliefs in Low Back Pain. *Spine (Phila. Pa. 1976).* **2006**, *31*, 1038–1046, doi:10.1097/01.brs.0000214878.01709.0e.
32.	Swinkels-Meewisse, I.E.; Roelofs, J.; Schouten, E.G.; Verbeek, A.L.; Oostendorp, R.A.; Vlaeyen, J.W. Fear of Movement/(Re)Injury Predicting Chronic Disabling Low Back Pain: A Prospective Inception Cohort Study. *Spine (Phila. Pa. 1976).* **2006**, *31*, 658–664, doi:10.1097/01.brs.0000203709.65384.9d.
33.	Van Der Windt, D.A.; Kuijpers, T.; Jellema, P.; Van Der Heijden, G.J.; Bouter, L.M. Do Psychological Factors Predict Outcome in Both Low-Back Pain and Shoulder Pain? *Ann. Rheum. Dis.* **2007**, *66*, 313–319, doi:10.1136/ard.2006.053553.
34.	Truchon, M.; Côté, D.; Schmouth, M.È.; Leblond, J.; Fillion, L.; Dionne, C. Validation of an Adaptation of the Stress Process Model for Predicting Low Back Pain Related Long-Term Disability Outcomes: A Cohort Study. *Spine (Phila. Pa. 1976).* **2010**, *35*, 1307–1315, doi:10.1097/BRS.0b013e3181c03d06.
35.	Soucy, I.; Truchon, M.; Côté, D. Work-Related Factors Contributing to Chronic Disability in Low Back Pain. *Work* **2006**, *26*, 313–326.
36.	Schultz, I.Z.; Crook, J.; Meloche, G.R.; Berkowitz, J.; Milner, R.; Zuberbier, O.A.; Meloche, W. Psychosocial Factors Predictive of Occupational Low Back Disability: Towards Development of a Return-to-Work Model. *Pain* **2004**, *107*, 77–85, doi:10.1016/j.pain.2003.09.019.
37.	Schultz, I.Z.; Crook, J.; Berkowitz, J.; Milner, R.; Meloche, G.R. Predicting Return to Work after Low Back Injury Using the Psychosocial Risk for Occupational Disability Instrument: A Validation Study. *J. Occup. Rehabil.* **2005**, *15*, 365–376, doi:10.1007/s10926-005-5943-9.
38.	Steenstra, I.A.; Koopman, F.S.; Knol, D.L.; Kat, E.; Bongers, P.M.; De Vet, H.C.W.; Van Mechelen, W. Prognostic Factors for Duration of Sick Leave Due to Low-Back Pain in Dutch Health Care Professionals. In Proceedings of the Journal of Occupational Rehabilitation; December 2005; Vol. 15, pp. 591–605.
39.	Karjalainen, K.; Malmivaara, A.; Mutanen, P.; Pohjolainen, T.; Roine, R.; Hurri, H. Outcome Determinants of Subacute Low Back Pain. *Spine (Phila. Pa. 1976).* **2003**, *28*, 2634–2640, doi:10.1097/01.BRS.0000099097.61495.2E.
40.	Shaw, W.S.; Pransky, G.; Patterson, W.; Winters, T. Early Disability Risk Factors for Low Back Pain Assessed at Outpatient Occupational Health Clinics. *Spine (Phila. Pa. 1976).* **2005**, *30*, 572–580, doi:10.1097/01.brs.0000154628.37515.ef.

41.	Hagen, E.M.; Svensen, E.; Eriksen, H.R. *Predictors and Modifiers of Treatment Effect Influencing Sick Leave in Subacute Low Back Pain Patients*; 2005; Vol. 30;.
42.	Kapoor, S.; Shaw, W.S.; Pransky, G.; Patterson, W. Initial Patient and Clinician Expectations of Return to Work after Acute Onset of Work-Related Low Back Pain. *J. Occup. Environ. Med.* **2006**, *48*, 1173–1180, doi:10.1097/01.jom.0000243401.22301.5e.
43.	Dionne, C.E.; Bourbonnais, R.; Frémont, P.; Rossignol, M.; Stock, S.R.; Larocque, I. A Clinical Return-to-Work Rule for Patients with Back Pain. *CMAJ* **2005**, *172*, 1559–1567, doi:10.1503/cmaj.1041159.
44.	Lötters, F.; Burdorf, A. Prognostic Factors for Duration of Sickness Absence Due to Musculoskeletal Disorders. *Clin. J. Pain* **2006**, *22*, 212–221, doi:10.1097/01.ajp.0000154047.30155.72.
45.	Reme, S.E.; Hagen, E.M.; Eriksen, H.R. Expectations, Perceptions, and Physiotherapy Predict Prolonged Sick Leave in Subacute Low Back Pain. *BMC Musculoskelet. Disord.* **2009**, *10*, 1–9, doi:10.1186/1471-2474-10-139.
46.	Dasinger, L.K.; Krause, N.; Deegan, L.J.; Brand, R.J.; Rudolph, L. Physical Workplace Factors and Return to Work After Compensated Low Back Injury: *J. Occup. Environ. Med.* **2000**, *42*, 323–333, doi:10.1097/00043764-200003000-00015.
47.	Wessels, T.; Ewert, T.; Limm, H.; Rackwitz, B.; Stucki, G. Change Factors Explaining Reductions of "Interference" in a Multidisciplinary and an Exercise Prevention Program for Low Back Pain. *Clin. J. Pain* **2007**, *23*, 629–634, doi:10.1097/AJP.0b013e31812e0060.
48.	Hill, J.C.; Dunn, K.M.; Lewis, M.; Mullis, R.; Main, C.J.; Foster, N.E.; Hay, E.M. A Primary Care Back Pain Screening Tool: Identifying Patient Subgroups for Initial Treatment. *Arthritis Care Res.* **2008**, *59*, 632–641, doi:10.1002/art.23563.
49.	Leeuw, M.; Goossens, M.E.; van Breukelen, G.J.; de Jong, J.R.; Heuts, P.H.; Smeets, R.J.; Köke, A.J.; Vlaeyen, J.W. Exposure in Vivo versus Operant Graded Activity in Chronic Low Back Pain Patients: Results of a Randomized Controlled Trial. *Pain* **2008**, *138*, 192–207, doi:10.1016/j.pain.2007.12.009.
50.	Kovacs, F.M.; Seco, J.; Royuela, A.; Corcoll-Reixach, J.; Peña-Arrebola, A. The Prognostic Value of Catastrophizing for Predicting the Clinical Evolution of Low Back Pain Patients: A Study in Routine Clinical Practice within the Spanish National Health Service. *Spine J.* **2012**, *12*, 545–555, doi:10.1016/j.spinee.2012.06.002.
51.	Foster, N.E.; Thomas, E.; Bishop, A.; Dunn, K.M.; Main, C.J. Distinctiveness of Psychological Obstacles to Recovery in Low Back Pain Patients in Primary Care. *Pain* **2010**, *148*, 398–406, doi:10.1016/j.pain.2009.11.002.
52.	Pinto, R.Z.; Ferreira, P.H.; Kongsted, A.; Ferreira, M.L.; Maher, C.G.; Kent, P. Self-Reported Moderate-to-Vigorous Leisure Time Physical Activity Predicts Less Pain and Disability over 12 Months in Chronic and Persistent Low Back Pain. *Eur. J. Pain (United Kingdom)* **2014**, *18*, 1190–1198, doi:10.1002/j.1532-2149.2014.00468.x.
53.	Huijnen, I.P.; Kindermans, H.P.; Seelen, H.A.; Peters, M.L.; Smeets, R.J.; Serroyen, J.; Roelofs, J.; Goossens, M.; Verbunt, J.A. Effects of Self-Discrepancies on Activity-Related Behaviour: Explaining Disability and Quality of Life in Patients with Chronic Low Back Pain. *Pain* **2011**, *152*, 2165–2172, doi:10.1016/j.pain.2011.05.028.
54.	Nordeman, L.; Thorselius, L.; Gunnarsson, R.; Mannerkorpi, K. Predictors for Future Activity Limitation in Women with Chronic Low Back Pain Consulting Primary Care: A 2-Year Prospective Longitudinal Cohort Study. *BMJ Open* **2017**, *7*, 1–11, doi:10.1136/bmjopen-2016-013974.
55.	Unsgaard-Tøndel, M.; Nilsen, T.I.L.; Magnussen, J.; Vasseljen, O. Is Activation of Transversus Abdominis and Obliquus Internus Abdominis Associated with Long-Term Changes in Chronic Low Back Pain? A Prospective Study with 1-Year Follow-Up. *Br. J. Sports Med.* **2012**, *46*, 729–734, doi:10.1136/bjsm.2011.085506.
56.	Heinrich, M.; Hafenbrack, K.; Michel, C.; Monstadt, D.; Marnitz, U.; Klinger, R. Measures of Success in Treatment of Chronic Back Pain: Pain Intensity, Disability and Functional Capacity: Determinants of Treatment Success in Multimodal Day Clinic Setting. *Schmerz* **2011**, *25*, 282–289, doi:10.1007/s00482-011-1027-2.
57.	Heymans, M.W.; Ford, J.J.; McMeeken, J.M.; Chan, A.; De Vet, H.C.; Van Mechelen, W. Exploring the Contribution of Patient-Reported and Clinician Based Variables for the Prediction of Low Back Work Status. *J. Occup. Rehabil.* **2007**, *17*, 383–397, doi:10.1007/s10926-007-9084-1.
58.	Dozois, D.J.; Dobson, K.S.; Wong, M.; Hughes, D.; Long, A. Predictive Utility of the CSQ in Low Back Pain: Individual vs. Composite Measures. *Pain* **1996**, *66*, 171–180, doi:10.1016/0304-3959(96)03058-8.
59.	Chibnall, J.T.; Tait, R.C. Long-Term Adjustment to Work-Related Low Back Pain: Associations with Socio-Demographics, Claim Processes, and Post-Settlement Adjustment. *Pain Med.* **2009**, *10*, 1378–1388, doi:10.1111/j.1526-4637.2009.00738.x.
60.	Hussain, S.M.; Urquhart, D.M.; Wang, Y.; Dunstan, D.; Shaw, J.E.; Magliano, D.J.; Wluka, A.; Cicuttini, F.M. Associations between Television Viewing and Physical Activity and Low Back Pain in Community-Based Adults: A Cohort Study. *Medicine (Baltimore).* **2016**, *95*, e3963, doi:10.1097/MD.0000000000003963.
61.	Kovacs, F.M.; Muriel, A.; Castillo Sánchez, M.D.; Medina, J.M.; Royuela, A.; Ruíz, J.L.; Fernández, C.; Olabe, J.; Gómez, P.I.; Abraira, V.; et al. Fear Avoidance Beliefs Influence Duration of Sick Leave in Spanish Low Back Pain Patients. *Spine (Phila. Pa. 1976).* **2007**, *32*, 1761–1766, doi:10.1097/BRS.0b013e3180b9f5f7.
62.	Helmhout, P.H.; Staal, J.B.; Heymans, M.W.; Harts, C.C.; Hendriks, E.J.M.; De Bie, R.A. Prognostic Factors for Perceived Recovery or Functional Improvement in Non-Specific Low Back Pain: Secondary Analyses of Three Randomized Clinical Trials. *Eur. Spine J.* **2010**, *19*, 650–659, doi:10.1007/s00586-009-1254-8.
63.	Jensen, O.K.; Nielsen, C.V.; Stengaard-Pedersen, K. One-Year Prognosis in Sick-Listed Low Back Pain Patients with and without Radiculopathy. Prognostic Factors Influencing Pain and Disability. *Spine J.* **2010**, *10*, 659–675, doi:10.1016/j.spinee.2010.03.026.
64.	van den Heuvel, S.G.; Ariëns, G.A.; Boshuizen, H.C.; Hoogendoorn, W.E.; Bongers, P.M. Prognostic Factors Related to Recurrent Low-Back Pain and Sickness Absence. *Scand. J. Work. Environ. Heal.* **2004**, *30*, 459–467, doi:10.5271/sjweh.835.
65.	Dionne, C.E.; Bourbonnais, R.; Frémont, P.; Rossignol, M.; Stock, S.R.; Nouwen, A.; Larocque, I.; Demers, E. Determinants of "Return to Work in Good Health" among Workers with Back Pain Who Consult in Primary Care Settings: A 2-Year Prospective Study. *Eur. Spine J.* **2007**, *16*, 641–655, doi:10.1007/s00586-006-0180-2.
66.	Gheldof, E.L.; Vinck, J.; Van den Bussche, E.; Vlaeyen, J.W.; Hidding, A.; Crombez, G. Pain and Pain-Related Fear Are Associated with Functional and Social Disability in an Occupational Setting: Evidence of Mediation by Pain-Related Fear. *Eur. J. Pain* **2006**, *10*, 513–525, doi:10.1016/j.ejpain.2005.07.005.
67.	Karlsson, N.; Skargren, E.; Kristenson, M. Emotional Support Predicts More Sickness Absence and Poorer Self Assessed Work Ability: A Two-Year Prospective Cohort Study. *BMC Public Health* **2010**, *10*, 648, doi:10.1186/1471-2458-10-648.
68.	Tubach, F.; Leclerc, A.; Landre, M.F.; Pietri-Taleb, F. Risk Factors for Sick Leave Due to Low Back Pain: A Prospective Study. *J. Occup. Environ. Med.* **2002**, *44*, 451–458, doi:10.1097/00043764-200205000-00013.
69.	Van Der Giezen, A.M.; Bouter, L.M.; Nijhuis, F.J.N. Prediction of Return-to-Work of Low Back Pain Patients Sicklisted for 3- 4 Months. *Pain* **2000**, *87*, 285–294, doi:10.1016/S0304-3959(00)00292-X.
70.	Bishop, F.L.; Yardley, L.; Prescott, P.; Cooper, C.; Little, P.; Lewith, G.T. Psychological Covariates of Longitudinal Changes in Back-Related Disability in Patients Undergoing Acupuncture. *Clin. J. Pain* **2015**, *31*, 254–264, doi:10.1097/AJP.0000000000000108.
71.	Goldstein, M.S.; Morgenstern, H.; Hurwitz, E.L.; Yu, F. The Impact of Treatment Confidence on Pain and Related Disability among Patients with LBP. *Spine J* **2002**, *2*, 391–399, doi:10.1016/s1529-9430(02)00414-x.
72.	Myers, S.S.; Phillips, R.S.; Davis, R.B.; Cherkin, D.C.; Legedza, A.; Kaptchuk, T.J.; Hrbek, A.; Buring, J.E.; Post, D.; Connelly, M.T.; et al. Patient Expectations as Predictors of Outcome in Patients with Acute Low Back Pain. *J. Gen. Intern. Med.* **2008**, *23*, 148–153, doi:10.1007/s11606-007-0460-5.
73.	Macedo, L.G.; Maher, C.G.; Hancock, M.J.; Kamper, S.J.; McAuley, J.H.; Stanton, T.R.; Stafford, R.; Hodges, P.W. Predicting Response to Motor Control Exercises and Graded Activity for Patients With Low Back Pain: Preplanned Secondary Analysis of a Randomized Controlled Trial. *Phys. Ther.* **2014**, *94*, 1543–1554, doi:10.2522/ptj.20140014.
74.	Sherman, K.J.; Cherkin, D.C.; Ichikawa, L.; Avins, A.L.; Barlow, W.E.; Khalsa, P.S.; Deyo, R.A. Characteristics of Patients with Chronic Back Pain Who Benefit from Acupuncture. *BMC Musculoskelet. Disord.* **2009**, *10*, 1–10, doi:10.1186/1471-2474-10-114.
75.	Tran, H.H.; Weinberg, J.; Sherman, K.J.; Saper, R.B. Preference and Expectation for Treatment Assignment in a Randomized Controlled Trial of Once- vs Twice-Weekly Yoga for Chronic Low Back Pain. *Glob. Adv. Heal. Med.* **2015**, *4*, 34–39, doi:10.7453/gahmj.2014.066.
76.	Underwood, M.R.; Morton, V.; Farrin, A. Do Baseline Characteristics Predict Response to Treatment for Low Back Pain? Secondary Analysis of the UK BEAM Dataset [ISRCTN32683578]. *Rheumatology* **2007**, *46*, 1297–1302, doi:10.1093/rheumatology/kem113.
77.	Butler, R.J.; Johnson, W.G.; Gray, B.P. Timing Makes a Difference: Early Nurse Case Management Intervention and Low Back Pain. *Prof. Case Manag.* **2007**, *12*, 316–329, doi:https://doi.org/10.1097/01.PCAMA.0000300404.07948.86.
78.	Carriere, J.; Thibault, P.; Sullivan, M. The Mediating Role of Recovery Expectancies on the Relation Between Depression and Return-to-Work. *J. Occup.*

	Rehabil. **2015**, *25*, 348–356, doi:10.1007/s10926-014-9543-4.
79.	Jensen, O.K.; Stengaard-Pedersen, K.; Jensen, C.; Nielsen, C.V. Prediction Model for Unsuccessful Return to Work after Hospital-Based Intervention in Low Back Pain Patients. *BMC Musculoskelet. Disord.* **2013**, *14*, 1–16, doi:10.1186/1471-2474-14-140.
80.	Lindell, O.; Johansson, S.E.; Strender, L.E. Predictors of Stable Return-to-Work in Non-Acute, Non-Specific Spinal Pain: Low Total Prior Sick-Listing, High Self Prediction and Young Age. A Two-Year Prospective Cohort Study. *BMC Fam Pr.* **2010**, *11*, 1–12, doi:10.1186/1471-2296-11-53.
81.	Magnussen, L.; Strand, L.I.; Skouen, J.S.; Eriksen, H.R. Motivating Disability Pensioners with Back Pain to Return to Work - A Randomized Controlled Trial. *J. Rehabil. Med.* **2007**, *39*, 81–87, doi:10.2340/16501977-0004.
82.	Opsahl, J.; Eriksen, H.R.; Tveito, T.H. Do Expectancies of Return to Work and Job Satisfaction Predict Actual Return to Work in Workers with Long Lasting LBP? *BMC Musculoskelet. Disord.* **2016**, *17*, 1–10, doi:10.1186/s12891-016-1314-2.
83.	Petersen, T.; Larsen, K.; Jacobsen, S. One-Year Follow-up Comparison of the Effectiveness of McKenzie Treatment and Strengthening Training for Patients with Chronic Low Back Pain: Outcome and Prognostic Factors. *Spine (Phila. Pa. 1976).* **2007**, *32*, 2948–2956, doi:10.1097/BRS.0b013e31815cda4a.
84.	Reiso, H.; Nygård, J.F.; Jørgensen, G.S.; Holanger, R.; Soldal, D.; Bruusgaard, D. Back to Work: Predictors of Return to Work among Patients with Back Disorders Certified as Sick: A Two-Year Follow-up Study. *Spine (Phila. Pa. 1976).* **2003**, *28*, 1468–1473, doi:10.1097/01.BRS.0000067089.83472.1F.
85.	Downie, A.S.; Maher, C.G.; Lin, C.-W.C.; Hancock, M.J.; Rzewuska, M.; Williams, C.M. Trajectories of Acute Low Back Pain: A Latent Class Growth Analysis. *Pain* **2016**, *157*, 225–234, doi:10.1097/j.pain.0000000000000351.
86.	Enthoven, P.; Skargren, E.; Carstensen, J.; Oberg, B. Predictive Factors for 1-Year and 5-Year Outcome for Disability in a Working Population of Patients with Low Back Pain Treated in Primary Care. *Pain* **2006**, *122*, 137–144, doi:10.1016/j.pain.2006.01.022.
87.	Foster, N.E.; Bishop, A.; Thomas, E.; Main, C.; Horne, R.; Weinman, J.; Hay, E. Illness Perceptions of Low Back Pain Patients in Primary Care: What Are They, Do They Change and Are They Associated with Outcome? *Pain* **2008**, *136*, 177–187, doi:10.1016/j.pain.2007.12.007.
88.	Henschke, N.; Maher, C.G.; Refshauge, K.M.; Herbert, R.D.; Cumming, R.G.; Bleasel, J.; York, J.; Das, A.; McAuley, J.H. Prognosis in Patients with Recent Onset Low Back Pain in Australian Primary Care: Inception Cohort Study. *Bmj* **2008**, *337*, 154–157, doi:10.1136/bmj.a171.
89.	Niemistö, L.; Sarna, S.; Lahtinen-Suopanki, T.; Lindgren, K.A.; Hurri, H. Predictive Factors for 1-Year Outcome of Chronic Low Back Pain Following Manipulation, Stabilizing Exercises, and Physician Consultation or Physician Consultation Alone. *J. Rehabil. Med.* **2004**, *36*, 104–109, doi:10.1080/16501970310019151.
90.	Rasmussen-Barr, E.; Campello, M.; Arvidsson, I.; Nilsson-Wikmar, L.; Äng, B.O. Factors Predicting Clinical Outcome 12 and 36 Months after an Exercise Intervention for Recurrent Low-Back Pain. *Disabil. Rehabil.* **2012**, *34*, 136–144, doi:10.3109/09638288.2011.591886.
91.	Reeser, J.C.; Wiegmann, S.M.; Hoover, N.; Oldridge, N.; Phillips, D.; Bjelland, T.; Scarpinato, L.; Treacy, W.; Helstad, C.P.; Stoll, J. Treatment of Acute Low Back Pain in Wisconsin: Results of the State Medical Society's Medical Outcomes Research Project. *Wis. Med. J.* **2001**, *100*, 35–42.
92.	Shaw, W.S.; Pransky, G.; Winters, T. The Back Disability Risk Questionnaire for Work-Related, Acute Back Pain: Prediction of Unresolved Problems at 3-Month Follow-Up. *J. Occup. Environ. Med.* **2009**, *51*, 185–194, doi:10.1097/JOM.0b013e318192bcf8.
93.	Yelland, M.J.; Schluter, P.J. Defining Worthwhile and Desired Responses to Treatment of Chronic Low Back Pain. *Pain Med.* **2006**, *7*, 38–45, doi:10.1111/j.1526-4637.2006.00087.x.
94.	Jensen, I.B.; Bodin, L.; Ljungqvist, T.; Gunnar Bergström, K.; Nygren, Å. Assessing the Needs of Patients in Pain: A Matter of Opinion? *Spine (Phila. Pa. 1976).* **2000**, *25*, 2816–2823, doi:10.1097/00007632-200011010-00015.
95.	Gervais, S.; Dupuis, G.; Véronneau, F.; Bergeron, Y.; Millette, D.; Avard, J. Predictive Model to Determine Cost/Benefit of Early Detection and Intervention in Occupational Low Back Pain. *J. Occup. Rehabil.* **1991**, *1*, 113–131, doi:10.1007/BF01073382.
96.	Morlock, R.J.; Lafata, J.E.; Nerenz, D.; Schiller, M.; Rosenblum, M. Expectations, Outcomes, and Medical Costs in Patients with Low Back Pain Referred to Physical Therapy. *Dis. Manag.* **2002**, *5*, 185–188, doi:10.1089/10935070260474958.
97.	Mahmud, M.A.; Webster, B.S.; Courtney, T.K.; Matz, S.; Tacci, J.A.; Christiani, D.C. Clinical Management and the Duration of Disability for Work-Related Low Back Pain. *J. Occup. Environ. Med.* **2000**, *42*, 1178–1187, doi:10.1097/00043764-200012000-00012.
98.	Burdorf, A.; Naaktgeboren, B.; Post, W. Prognostic Factors for Musculoskeletal Sickness Absence and Return to Work among Welders and Metal Workers. *Occup. Environ. Med.* **1998**, *55*, 490–495, doi:10.1136/oem.55.7.490.
99.	Hendrick, P.; Milosavljevic, S.; Hale, L.; Hurley, D.A.; McDonough, S.M.; Herbison, P.; Baxter, G.D. Does a Patient's Physical Activity Predict Recovery from an Episode of Acute Low Back Pain? A Prospective Cohort Study. *BMC Musculoskelet. Disord.* **2013**, *14*, 126, doi:10.1186/1471-2474-14-126.
100.	Prkachin, K.M.; Schultz, I.Z.; Hughes, E. Pain Behavior and the Development of Pain-Related Disability: The Importance of Guarding. *Clin J Pain* **2007**, *23*, 270–277, doi:10.1097/AJP.0b013e3180308d28.
101.	Flynn, T.; Fritz, J.; Whitman, J.; Wainner, R.; Magel, J.; Rendeiro, D.; Butler, B.; Garber, M.; Allison, S. A Clinical Prediction Rule for Classifying Patients with Low Back Pain Who Demonstrate Short-Term Improvement With Spinal Manipulation. *Spine (Phila. Pa. 1976).* **2002**, *27*, 2835–2843, doi:10.1097/00007632-200212150-00021.
102.	Roland, M.; Morris, R. A Study of the Natural History of Low-Back Pain. Part II: Development of Guidelines for Trials of Treatment in Primary Care. *Spine (Phila. Pa. 1976).* **1983**, *8*, 145–150, doi:10.1097/00007632-198303000-00005.
103.	Pedersen, P.A. Prognostic Indicators in Low Back Pain. *J. R. Coll. Gen. Pract.* **1981**, *31*, 209–216.
104.	Franklin, G.M.; Rahman, E.A.; Turner, J.A.; Daniell, W.E.; Fulton-Kehoe, D. *Opioid Use for Chronic Low Back Pain A Prospective, Population-Based Study Among Injured Workers in Washington State, 2002-2005*; 2009; Vol. 25;.
105.	Pransky, G.S.; Verma, S.K.; Okurowski, L.; Webster, B. Length of Disability Prognosis in Acute Occupational Low Back Pain. *Spine (Phila. Pa. 1976).* **2006**, *31*, 690–697, doi:10.1097/01.brs.0000202761.20896.02.
106.	Jellema, P.; van der Windt, D.A.W.M.; van der Horst, H.E.; Stalman, W.A.B.; Bouter, L.M. Prediction of an Unfavourable Course of Low Back Pain in General Practice: Comparison of Four Instruments. *Br. J. Gen. Pract.* **2007**, *57*, 15–22.
107.	Valencia, C.; Robinson, M.E.; George, S.Z. Socioeconomic Status Influences the Relationship between Fear-Avoidance Beliefs Work and Disability. *Pain Med.* **2011**, *12*, 328–336, doi:10.1111/j.1526-4637.2010.01024.x.
108.	Lanier, D.C.; Stockton, P. Clinical Predictors of Outcome of Acute Episodes of Low Back Pain. *J. Fam. Pract.* **1988**, *27*, 483–489.
109.	Reis, S.; Hermoni, D.; Borkan, J.M.; Biderman, A.; Tabenkin, C.; Porat, A. A New Look at Low Back Complaints in Primary Care: A RAMBAM Israeli Family Practice Research Network Study. *J. Fam. Pract.* **1999**, *48*, 299–303.

Supplementary Table S6: Other statistics (RR, RP, HR, LR+LR- and p values)

Supplementary Table S6.1 Prognostic factors for acute, subacute, chronic and mixed-duration LBP outcomes at short- and long-term using RR/PR

Prognostic factor domain	Prognostic factor	Factor definition	Author, year [Ref]	Nº primary studies included (N)	Outcome	Follow-up	Ajusted RR/RP	Crude RR/RP
**Acute LBP (<6 weeks)**								
**Occupational**	Work psychosocial factors	Workplace psychosocial factors (greater psychosocial stress)	Steenstra IA, 2011 [28]	1 study (N=433)	WP	NR	RR ranged from 0.85, 95%CI= 0.65-1.13 to 1.04, 95%CI= 0.77-1.41	
**Socio-occupational**	Work social support	Supervisor support (Low)	Steenstra IA, 2011 [28]	1 study (N=433)	WP	NR	Unclear	
**Acute and subacute LBP (≤3 months)**								
**Psychological-cognitive**	Pain catastrophism	Pan catastrophism (High) (PCS, CSQ, PRSS)	Wertli MM, 2014 [36]	1 study (N=253)	WP	ST		PR= 1.01, 95%CI= 0.98–1.04
						LT		PR= 1.01, 95%CI= 0.95–1.07
**Chronic LBP (>3 months)**								
**General health**	Physical activity	High self-reported physical activity	Oliveira CB, 2019 [40]	1 study (N=1,836)	Ra	LT	RR women= **1.66, 95% CI= 1.07-2.55**<br>RR men= 1.06 (95% CI= 0.66 to 1.71)	
**Acute to chronic LBP**								
**Psychological-cognitive**	Fear avoidance beliefs	High fear avoidance beliefs (TSK)	Wertli MM, 2014 [35]	1 study (N=389)	Ra	LT	RR= 0.46, p >0,05	
	Pain catastrophism	Pan catastrophism (High) (CSQ)	Wertli MM, 2014 [36]	1 study (N=389)	FS	LT	RR= 1.46, 95%CI= 0.83–2.54	
**Socio-occupational**	Co-worker social support	Co-worker support (high)	Campbell P, 2013 [30]	1 study (ND)	Rd*	ST	RR= **1.55, 95%CI= 1.04- 2.34**	
	Supervisor social suppor	Supervisor support (high)	Campbell P, 2013 [30]	1 study (ND)	Rd*	ST	RR= 0.71, 95%CI= 0.34-1.48	

LBP= Low back pain; N= sample size; RR= Risk ratio; PR= Prevalence ratio; LT= Long-term follow up (≥ 3 months); ST= Sort-term follow-up (<3 months); NR= non reported

Outcome: FS= Functional status; WP= Work participation; R (a-d): Recovery a= recovery of pain or disability, d= recovery and/or return to work

*Associated with good outcomes

Bold results are statistically significant

Supplementary Table S6.2 Prognostic factors for acute, subacute, chronic and mixed-duration LBP outcomes at short- and long-term using HR

Prognostic factor domain	Prognostic factor	Factor definition	Author, year [Ref]	Nº primary studies included (N)	Outcome	Adjusted HR	Crude HR
**Acute LBP (<6 weeks)**							
**Individual related factots**	Presence of language barriers	Presence of language barriers (yes/no)	Steenstra IA, 2011 [27]	1 study (N= 494)	WP	HR= 1.54, 95% CI= 1.05-2.27	
**Treatment related factors**	Type of treatment	Treatment related factor- content (provider)	Steenstra IA, 2011 [27]	1 study (N= 615)	WP		Treatment GP or medical specialist, HR= 1.95, 95% CI= 1.59- 2.38. Seeking Occupational care, HR= 1.83, 95% CI= 1.32-2.50
**Pain**	Pain intensity	Pain intensity (high)	Steenstra IA, 2011 [27]	3 studies (N= 1,795)	WP	HR ranged from 1.17, 95% CI= 1.05–1.29 to 1.09, 95% CI= 0.68-1.75	HR ranged from 0.94, 95% CI= 0.90–0.98 to 1.12, 95% CI= 1.04-1.20
**Functional limitation**	Disability	High self-reported disability (RMDQ, ODI, others)	Steenstra IA, 2011 [27]	3 studies (N= 984)	WP	HR ranged from 1.22, 95% CI= 1.11-1.37 to 1.97, 95% CI= 1.45-2.70	
**Psychological-cognitive**	Fear avoidance beliefs	High fear avoidance beliefs	Steenstra IA, 2011 [27]	1 study (N= 628)	WP		HR= 0.98, 95% CI= 0.97–1.00
	Recovery expectations	Low recovery expectations (How likely it is they will return to work/how long it will be before they are able to return)	Steenstra IA, 2011 [27]	3 studies (N= 1,180)	WP	HR ranged from 0.95, 95% CI= 0.91-1.00 to 2.32, 95% CI= 1.29-3.33	HR= 0.95, 95% CI= 0.91–1.00
**Socio-occupational**	Claim related factors	Claim related factors (presence/absence) (compensation, health insurance, lawyer involved)	Steenstra IA, 2011 [27]	1 study (N= 494)	WP	Delayed nurse case manager referral, HR= **0.64, 95% CI= 0.42-0.98**	
	Job satisfaction	Job satisfaction (low) (All in all, were you satisfied with your job?)	Steenstra IA, 2011 [27]	2 studies (N= 927)	WP		HR ranged from 0.93, 95% CI= 0.79- 1.07 to 1.70, 95% CI= 1.06-2.70
**Acute and subacute LBP (≤3 months)**							
**Psychological-emotional**	Emotional distress	Symptoms of depression (high)	Pinheiro MB, 2016 [36]	1 study (N= 973)	Ra		HR= 0.94, 95% CI= 0.91–0.97
**Psychological-cognitive**	Recovery expectations	Recovery expectations (low)	Iles RA, 2009 [30]	1 study (N= 596)	WP		HR ranged from 2.83, 95%CI= 2.04–4.00 to 3.50, 95% CI= 2.27–3.03
**Chronic LBP (>3 months)**							
**Psychological-cognitive**	Fear avoidance beliefs	High fear avoidance beliefs (FABQ y TSK)	Wertli MM, 2014 [33]	1 study (N= 628)	WP	HR= 0.98, 95% CI= 0.97–1.0	
**Acute to chronic LBP**							
**Socio-occupational**	General work support	General work support (low)	Campbell P, 2013 [29]	9 studies (ND)	Rd	HR ranged from 1.04, 95% CI= 1.0-1.08 to 1.05, 95% CI= 0.86–1.28	

LBP= Low back pain; N= sample size; HR= Hazard ratio

Outcome: P= Pain; FS= Functional status; WP= Work participation; R (a b c d): Recovery a= recovery of pain or disability, b= self-reported recovery, c= slightly better" or "worse" score on two or more follow-up measurements, d= recovery and/or return to work

Bold results are statistically significant

Supplementary Table S6.3 Prognostic factors for acute LBP outcomes at short- and long- term using LR+/LR-

Prognostic factor domain	Prognostic factor	Factor definition	Author, year [Ref]	Nº primary studies included (N)	Outcome	Follow-up	LR+ / LR-
**Acute LBP (<8 weeks)**							
**Individual related factots**	Age	Age (≤40, <45, or <46 years vs older)	Chou R, 2010 [26]	6 studies (N=3,746)	Rd	ST	LR positive= median 0.94 (range 0.74-1.1)/ LR negative= median 1.1 (range 0.81-2.0)
				6 studies (N=4,541)	Rd	LT	LR positive= median 0.93 (range 0.62-1.0)/ LR negative= median 1.1 (range 0.99-1.8)
	Gender	Sex (Female vs male)	Chou R, 2010 [26]	9 studies (N=4,709)	Rd	ST	LR positive= median 1.1 (range 0.72-1.4)/ LR negative= median 0.94 (range 0.66-1.3)
				8 studies (N=4,384)	Rd	LT	LR positive= median 1.3 (range 1.0-1.7)/ LR negative= median 0.73 (range 0.58-1.0)***
	Education	Education (no college or graduate education vs. more education)	Chou R, 2010 [26]	7 studies (N=4,150)	Rd	ST	LR positive= median 1.0 (range 0.97-1.3)/ LR negative= median 0.76 (range 0.52-1.1)
				4 studies (N=3,858)	Rd	LT	LR positive = median 1.1 (range 1.1-1.2)/ LR negative = median 0.65 (range 0.46-0.85)
**General health**	BMI	Overweight (BMI >25 o ≥27 vs low BMI)	Chou R, 2010 [26]	3 studies (N=2,237)	Rd	ST	LR positive: median 0.91 (range 0.72-1.2)/ LR negative: median 1.0 (range 0.76-1.2)
				2 studies (N=1,383)	Rd	LT	LR positive: median 0.84 (range 0.73-0.97)/ LR negative: median 1.1 (range 1.0-1.2)
	General health	General health status or activity level before the onset of the low back pain (lower vs. better)	Chou R, 2010 [26]	3 studies (N=2,174)	Rd	ST	LR positive : median **1.6 (range 1.1-1.7)** / LR negative: median **0.73 (range 0.66-0.88)**
				5 studies (N=4,137)	Rd	LT	LR positive: median **1.8 (range 1.1-2.0)**/ LR negative: median **0.85 (range 0.56-0.99)**
	Mental health	Psychiatric comorbidities (major vs. minor)	Chou R, 2010 [26]	4 studies (N=3,123)	Rd	ST	LR positive: median **1.9 (range 1.4-2.1)**/ LR negative: median **0.69 (range 0.55-0.85)**
				4 studies (N=3,957)	Rd	LT	LR positive: median **2.2 (range 1.9-2.3)**/ LR negative: median **0.85 (range 0.55-0.93)**
	Smoking	Smoking status (current smoker vs. current non-smoker)	Chou R, 2010 [26]	3 studies (N=1,854)	Rd	ST	LR positive: median 1.2 (range 1.0-1.6)/ LR negative: median 0.88 (range 0.71-0.97)
**Episode related factors**	Previous history of low back pain	Previous episodes of low back pain (more episodes vs less or none)	Chou R, 2010 [26]	6 studies (N=2,629)	Rd	ST	LR positive: median 1.0 (range 0.90-1.2)/ LR negative: median 0.88 (range 0.53-1.1)***
				5 studies (N=2,405)	Rd	LT	LR positive: median 1.1 (range 0.95-1.2)/ LR negative: median 0.81 (range 0.32-1.1)***
**Pain**	Pain radiating to the leg	Radiculopathy or leg pain (Y/N)	Chou R, 2010 [26]	5 studies (N=2,824)	Rd	ST	LR positive: median **1.4 (range 1.1-1.7)**/ LR negative: median **0.63 (range 0.52-0.93)**
				7 studies (N=4,760)	Rd	LT	LR positive: median **1.4 (range 1.2-2.4)**/ LR negative: median **0.82 (range 0.54-0.94)**
	Pain intensity	Basal Pain (High-Middle-Low Pain Intensity)	Chou R, 2010 [26]	6 studies (N=3,402)	Rd	ST	LR positive: median **1.7 (range 1.1-3.7)**/ LR negative: median **0.70 (range 0.07-0.86)*****
				3 studies (N=3,110)	Rd	LT	LR positive : median **1.3 (range 1.2-2.0)**/ LR negative: median **0.33 (range 0.08-0.97)*****
**Functional limitation**	Disability	Basal function (high-medium-low functional impairment category: RMDQ, ODI and other ordinal scales)	Chou R, 2010 [26]	6 studies (N=4,030)	Rd	ST	LR positive: median **1.4 (range 1.3-3.5)**/ LR negative: median **0.53 (range 0.18-1.1)**
				3 studies (N=3,738)	Rd	LT	LR positive: median **2.1 (range 1.2-2.7)**/ LR negative: median **0.40 (range 0.10-0.52)**
**Psychological-emotional**	Somatization	Waddell's non-organic signs (somatization) (more vs. less)	Chou R, 2010 [26]	1 study (N=880)	Rd	ST	LR positive: **2.5 (IC 95% 1.8-3.4)**/ LR negative: **0.81 (IC 95% 0.74-0.89)**
				3 studies (N=2,945)	Rd	LT	LR positive: median **3.0 (range 1.7-4.6)**/ LR negative: median **0.71 (range 0.31-0.76)**
**Psychological-cognitive**	Fear avoidance behaviors/coping	Fear-avoidance/ coping behaviors (high-medium-low category: FABQ and others)	Chou R, 2010 [26]	4 studies (N=2,736)	Rd	ST	LR positive: median **2.2 (range 1.5-4.9)** / LR negative: median **0.46 (range 0.30-0.73)**
				2 studies (N=2,765)	Rd	LT	LR positive: median **2.5 (range 2.2-2.8)**/ LR negative: median **0.39 (range 0.38-0.40)**
**Occupational**	Work physical demands	Work physical demands (higher vs. lower)	Chou R, 2010 [26]	3 studies (N=2,174)	Rd	ST	LR positive: median 1.2 (range 1.1-1.6)/ LR negative: median 0.87 (range 0.85-0.89)
				2 studies (N=2,765)	Rd	LT	LR positive: median **1.4 (range 1.2-1.7)**/ LR negative: median **0.84 (range 0.83-0.85)**
	Compensation	Receive compensation at the basal level (sick leave, out of work, case of compensation of workers yes/no)	Chou R, 2010 [26]	4 studies (N=1,970)	Rd	ST	LR positive: median 1.3 (range 0.97-2.7)/ LR negative: median0.88 (range 0.78-1.0)
				5 studies (N=2,980)	Rd	LT	LR positive: median **1.4 (range 1.2-1.8)**/ LR negative: median **0.86 (range 0.37-0.93)*****
	Job satisfaction	Job satisfaction (lower vs. higher)	Chou R, 2010 [26]	3 studies (N=1,854)	Rd	ST	LR positive: median 1.1 (range 0.64-1.8)/ LR negative: median 0.98 (range 0.94-1.2)
				3 studies (N=1,180)	Rd	LT	LR positive: median **1.5 (range 1.3-1.8)**/ LR negative: median **0.88 (range 0.62-0.94)**
**Acute and subacute LBP (≤3 months)**							
**Psychological-cognitive**	Fear avoidance beliefs	High fear avoidance beliefs (FABQ y TSK)	Wertli MM, 2014 [33]	2 studies (N=331)	FS	LT	Unclear

LBP= Low back pain; N= sample size; LR= Likelihood ratio; LT= Long-term follow up (≥ 3 months); ST= Sort-term follow-up (<3 months)

Outcome: FS= Functional status; Rd= Recovery d (recovery and/or return to work)

*** Meta-analysis combining adjusted and adjusted data

Bold results are statistically significant

Supplementary Table S6.4 Prognostic factors for acute, subacute, chronic and mixed-duration LBP outcomes (p values)

Prognostic factor domain	Prognostic factor	Factor definition	Author, year [Ref]	Nº primary studies included (N)	Outcome	p value
**Acute LBP (<6 weeks)**						
**Pain**	Pain radiationg to the leg	Pain radiating to the leg	Steenstra IA, 2011 [27]	6 studies (N=4,245)	WP	**p= 0.0010**
	Higher pain intensity	Hiher pain intensity	Steenstra IA, 2011 [27]	9 studies (N=6,238)	WP	**p= 0.0010**
	Pain medication	Pain medication (opioid use)	Steenstra IA, 2011 [27]	1 study (N=8,443)	WP	Unclear
**Psychological-cognitive**	Recovery expectations	Lower recovery expectations (How likely it is they will return to work and/or how long it will be before they are able to return)	Steenstra IA, 2011 [27]	1 study (N=300)	WP	**p< 0.001**
**Occupational**	Alternative duties available	Alternative duties available	Steenstra IA, 2011 [27]	3 studies (N=2,586)	WP	Unclear
**Acute and subacute LBP (≤3 months)**						
**General health**	Physical activity	High physical activity (any type)	Oliveira CB, 2019 [38]	1 study (N=106)	Ra	p= 0,58
**Psychological-emotional**	Depression	High basal symptoms of depression	Pinheiro MB, 2016 [36]	1 study (N=91)	FS	p= 0.57
				3 studies (N=428)	Ra	Unclear
**Chronic LBP**						
**General health**	Physical activity	High physical activity (any type)	Oliveira CB, 2019 [38]	1 study (N=211)	FS	p= 0,76
**Acute to chronic LBP**						
**General health**	Physical activity	Perform daily physical activity - occupational, sports and leisure activities	Hendrick P, 2011 [36]	4 studies (N=3,002)	P	p= 0.14
				3 studies (N=1,714)	FS	Unclear
				1 study (N=352)	NT	p= 0.064
		High physical activity (any type)	Oliveira CB, 2019 [38]	2 studies (N=505)	P	p values ranged from 0.38 to 0.29
				3 studies (N=1,060)	FS	p values ranged from 0.28 to 0.96
**Psychological-cognitive**	Pain catastrophism (CSQ, PRSS, PCC)	High pain catastrophism(CSQ, PRSS, PCC)	Wertli MM, 2014 [35]	2 studies (N=335)	P	**p < 0.05**
				2 studies (N=335)	FS	**p < 0.05**
				1 study (N=165)	TS	p > 0.05

LBP= Low back pain; N= sample size

Outcome: P= Pain; FS= Functional status; WP= Work participation; Ra: Recovery a= recovery pain or disability; NT= Nº Medical Treatments for Low Back Pain; TS= Treatment satisfaction

Bold results are statistically significant

Supplementary Table S7: Reliability of included reviews

Review	A. Identification, selection and critical appraisal of studies[1]						B. Analysis[2]						C. Overall[3]	
	**1. Selection criteria**	**2. Search**	**3. Up-to-date**	**4. Study selection**	**5. Risk of bias**	**6. Overall**	**1. Study characteristics**	**2. Analytic methods**	**3. Heterogeneity**	**4. Appropriate synthesis**	**5. Exploratory factors**	**6. Overall**	**1. Other considerations**	**2. Reliability of the review**
Kent PM, 2008	(+)	?	(+)	?	(+)	(+)	?	(+)	(+)	(+)	(+)	(+)	?	(+)
Chou R, 2010	(+)	?	(+)	?	(+)	(+)	(+)	(+)	(+)	(+)	(+)	(+)	?	(+)
Steenstra IA, 2011	(+)	?	(+)	?	?	(+)	(+)	(+)	(+)	(+)	(+)	(+)	?	(+)
Agnello A, 2010	?	?	?	?	(+)	(+)	(+)	(+)	(+)	(+)	(+)	(+)	(+)	(+)
Campbell P, 2013	(+)	?	?	?	(+)	(+)	?	(+)	(+)	(+)	(+)	(+)	(+)	(+)
Hallegraeff JM, 2012	(+)	?	(-)	?	(+)	(+)	(+)	(+)	(+)	(+)	(+)	(+)	?	(+)
Iles RA, 2009	(+)	?	?	?	(+)	(+)	?	(+)	(+)	(+)	(+)	(+)	?	(+)
Wertli & Rasmussen-Barr, 2014	(+)	?	?	?	(+)	(+)	(+)	(+)	(+)	(+)	(+)	(+)	(+)	(+)
Wertli & Eugster, 2014	(+)	?	?	?	(+)	(+)	(+)	(+)	(+)	(+)	(+)	(+)	(+)	(+)
Wertli & Burgstaller, 2014	(+)	?	?	?	(+)	(+)	(+)	(+)	(+)	(+)	?	(+)	?	(+)
Pinheiro MB, 2016	(+)	?	?	?	(+)	(+)	(+)	(+)	(+)	(+)	(+)	(+)	(+)	(+)
Hendrick P, 2011	(+)	?	?	?	(+)	(+)	(+)	(+)	(+)	(+)	(+)	(+)	(+)	(+)
Oliveira CB, 2019	(+)	?	(+)	(+)	(+)	(+)	(+)	(+)	(+)	(+)	(+)	(+)	?	(+)
Wong AY, 2013	(+)	(+)	(+)	?	(+)	(+)	(+)	(+)	(+)	(+)	(+)	(+)	?	(+)
Hayden JA, 2019	(+)	(+)	(+)	(+)	(+)	(+)	(+)	(+)	(+)	(+)	(+)	(+)	(+)	(+)
**Total (+)**	**14**	**2**	**6**	**2**	**14**	**15**	**12**	**15**	**15**	**15**	**14**	**15**	**7**	**15**
**Total (-)**	**0**	**0**	**1**	**0**	**0**	**0**	**0**	**0**	**0**	**0**	**0**	**0**	**0**	**0**
**Total NA**	**0**	**0**	**0**	**0**	**0**	**0**	**0**	**0**	**0**	**0**	**0**	**0**	**0**	**0**
**Total ?**	**1**	**13**	**8**	**13**	**1**	**0**	**3**	**0**	**0**	**0**	**1**	**0**	**8**	**0**

[1]**A. Identification, selection and critical appraisal of studies**

1. **Selection criteria**: were the criteria used for deciding which studies to include in the review reported? (+ yes; ? can't tell/partially; − no)

2. **Search**: was the search for evidence reasonably comprehensive? (+ yes; ? can't tell/partially; − no)

3. **Up-to-date**: is the review reasonably up-to-date? (+ yes; ? can't tell/partially; − no)

4. **Study selection**: was bias in the selection of articles avoided? (+ yes; ? can't tell/partially; − no)

5. R**isk of bias**: did the authors use appropriate criteria to assess the risk for bias in analysing the studies that are included? (+ yes; ? can't tell/partially; − no)

6. Overall: how would you rate the methods used to identify, include and critically appraise studies? (+ only minor limitations, − important limitations)

[2]**B. Analysis**

1. **Study characteristics**: were the characteristics and results of the included studies reliably reported? (+ yes; ? can't tell/partially;− no, NA not applicable; e.g. no studies or data)

2. **Analytic methods**: were the methods used by the review authors to analyse the findings of the included studies reported? (+ yes; ? can't tell/partially; − no, NA not applicable; e.g. no studies or data)

3. **Heterogeneity**: did the review describe the extent of heterogeneity? (+ yes; ? can't tell/partially; − no, NA not applicable; e.g. no studies or data)

4. **Appropriate synthesis**: were the findings of the relevant studies combined (or not combined) appropriately relative to the primary question the review addresses and the available data? (+ yes; ? can't tell/partially; − no, NA not applicable; e.g. no studies or data)

5. **Exploratory factors**: did the review examine the extent to which specific factors might explain differences in the results of the included studies? (+ yes; ? can't tell/partially; − no, NA not applicable; e.g. no studies or data)

6. **Overall**: how would you rate the methods used to analyse the findings relative to the primary question addressed in the review? (+ only minor limitations, − important limitations)	
[3]**C. Overall**	
1. **Other considerations**: are there any other aspects of the review not mentioned before which lead you to question the results? (+ yes; ? can't tell/partially; − no)	
2. **Reliability of the review**: based on the above assessments of the methods how would you rate the reliability of the review? (+ only minor limitations, − important limitations)	

Supplementary Table S8: Prognostic factors reported by only one review

Table S8. Prognostic factors for acute and subacute LBP outcomes reported by a single systematic review using OR/Beta Coefficients

Prognostic factor domain	Prognostic factor	Factor definition	Author, year [Ref]	Nº primary studies included (N) [Ref]	Outcome	Follow-up	Ajusted	Crude
**Acute and subacute LBP (≤3 months)**								
**Individual related factors**	Age	Older age	Kent PM, 2008 [26]	2 studies (N=818) [9, 10]	FS	LT	Pooled OR= 2.23, 95% CI= 0.35-14.07	
				2 studies (N=1,034) [11, 19]	WP	LT	**Pooled OR= 1.56, 95% CI= 1.05-2.30**	
	Marital status	Marital status (married/other)	Kent PM, 2008 [26]	3 studies (N=9,436) [13, 14, 97]	WP	LT		Largest significant OR= **0.31, 95% CI= 0.15–0.65**
	Being a parent	Being a parent	Kent PM, 2008 [26]	2 studies (N=8,912) [13, 14]	WP	LT		Largest significant OR= **2.69, 95% CI= 1.09–6.66**
**General health**	BMI	Higher BMI	Kent PM, 2008 [26]	2 studies (N=1,378) [11, 9]	WP	LT	Largest significant OR= **1.85, 95% CI= 1.17–2.90** a	
	General health	Worst general health (self-reported)	Kent PM, 2008 [26]	1 study (N=219) [7]	P	LT	Largest significant OR= **2.22, 95% CI= 1.14–4.34**	
				2 studies (N=414) [10, 15]	FS	LT	Largest significant OR= **4.56, 95% CI= 2.19–9.49**	
		Worse General Health Questionnaire Score		1 study (N=294) [10]	FS	LT	Largest significant OR= **3.34, 95% CI= 1.50–7.20**	
				1 study (N=854) [11]	WP	LT	Largest significant OR= **2.78, 95% CI= 2.00–3.87**	
	Mental health	Mental Health (Worse than average population)	Steenstra IA, 2011 [28]*	4 studies (N=3,114) [5, 12, 37, 98]	WP	LT	OR ranged from 1.11, 95%CI= 0.66-1.87 to 1.84, 95%CI= 0.99-3.42.	Odds ratio ranged from 1.03 (0.99-1.07) to 1.03 (NR)
	Physical activity	Physical activity (any type) (more active)	Oliveira CB, 2019 [40]	1 study (N=101) [99]	FS	ST	β=0.00, 95% CI=−0.00- 0.00, p=0.20	
**Treatment related factors**	Previous therapy	Previous therapy (yes/no)	Kent PM, 2008 [26]	1 study (N=524) [9]	FS	LT	Largest significant OR= **2.12, 95% CI= 1.30–3.50**	
	Previous Rx	Previous Rx (yes/no)		1 study (N=524) [9]	FS	LT	Largest significant OR= **2.53, 95% CI= 1.60–4.10**	
	Diagnosis	Diagnosis exists (yes/no)		1 study (N=524) [9]	FS	LT	Largest significant OR= **11.6, 95% CI= 2.90–46.90**	
	Type of treatment	Type of treatment (type of health care provider)	Steenstra IA, 2011 [28]*	2 studies (N=2,001) [12, 15]	WP	LT	OR ranged from **0.41, 95%CI= 0.24-0.70** to **1.93, 95%CI= 1.31-2.84**	
	Clinical exam findings	Clinical exam findings (Abnormal heel walk)		1 studies (N=162) [21]	WP	NR	OR= **2.53, 95%CI= 1.18-5.41, p=0.02**	
	Pain observation	Observation of pain (protection against pain, non-organic Waddell's signs, symptoms inconsistent with clinical findings)		2 studies (N=259) [37, 100]	WP	LT		Odds ratio ranged from **1.14, p=0.01** to **1.69, 95%CI= 1.15-2.23**
**Pain**	Longer pain duration	Longer pain duration	Kent PM, 2008 [26]	4 studies (N=1,123) [9, 10, 101, 102]	FS	LT	Pooled OR= 2.27, 95% CI= 0.53-9.65	
	Unilateral pain	Unilateral pain (yes/no)	Kent PM, 2008 [26]	1 study (N= 78) [103]	FS	LT		Largest significant OR= **4.91, 95% CI= 1.62–14.83**
	Widespread pain	Widespread pain (si/no)	Kent PM, 2008 [26]	1 study (N=294) [10]	FS	LT	Largest significant OR= **6.42, 95% CI= 2.70–15.00**	
	Pain medication	Pain medication (nº of opioid prescriptions in 6 wk)	Steenstra IA, 2011 [28]*	3 studies (N=2,683) [20, 104, 105]	WP	LT	OR= **2.5, 95%CI= 1.4–4.3**	Unclear
**Physical impairment**	Spine flexion	Limited spine flexion	Kent PM, 2008 [26]	1 study (N=294) [10]	FS	LT	Largest significant OR= **4.49, 95% CI= 2.10–9.50**	
	Knee ROM	Limited knee extension	Kent PM, 2008 [26]	1 study (N=294) [10]	FS	LT	Largest significant OR= **2.39, 95% CI= 1.10–5.10**	
	SLR	SLR positivo	Kent PM, 2008 [26]	3 studies (N=832) [9, 102, 103]	FS	LT		Pooled OR= 4.24, 95% CI= 0.40–44.84
	Aerobic capacity	Greater aerobic capacity	Kent PM, 2008 [26]	1 study (N=294) [10]	FS	LT	Largest significant OR= **2.80, 95% CI= 1.40–5.60**	

	Cranio-sacral axial compression	Centralisation	Kent PM, 2008 [26]	1 study (N=300) [6]	WP	LT	Largest significant OR= **8.83, 95% CI= 1.94–40.11** a	
**Emotion psychological**	Somatization	Somatic anxiety (somatization)	Kent PM, 2008 [26]	2 studies (N=1,154) [6, 11]	WP	LT	Pooled OR= 1.41, 95% CI= 0.82–2.44	

Table S8 ***(continued)***

**Cognitive psychological**	Pain catastrophism	Pan catastrophism (High) (PCS, CSQ, PRSS)	Wertli MM, 2014 [36]*	1 study (N=171) [33]	P	LT	OR= **2.45, 95%CI= 1.09–5.5**	
				1 study (N=171) [33]	FS	LT	OR= 3.31, 95%CI= 0.93–44.9	
				1 study (N=314) [106]	Rc	LT	OR= 1.04, 95%CI= 0.99–1.09, p=0.09	
		Pan catastrophism (High) (PCS, CSQ, PRSS)	Wertli MM, 2014 [36]	1 study (N=108) [107]	P	LT	Beta= **0.31 (95% CI NR), p=0.004**	
				2 studies (N=366) [24, 107]	FS	LT	Beta coefficients ranged from **0.43, p=0.001** to **1.68, 95%CI= 0.01–3.36, p=0.051**	
				2 studies (N=2,231) [12, 20]	WP	LT	OR= 1.33, 95%CI= 0.71–2.48	
**Mixed psychological**	Coping strategies	Poor coping strategies	Kent PM, 2008 [26]	2 studies (N=644) [9, 15]	FS	LT	Pooled OR= **3.24, 95% CI= 1.46–7.22**	
	Locus of control	Locus of control (external)	Kent PM, 2008 [26]	1 study (N=120) [15]	FS	LT	Largest significant OR= **4.30, 95% CI= 1.60–12.00**	
**Occupational**	Work status	Increased time away from work	Kent PM, 2008 [26]	4 studies (N=3,112) [30, 103, 108, 109]	FS	LT		Largest significant OR= **15.1, 95% CI= 4.64–49.17**
				2 studies (N=480) [6, 19]	WP	LT	Pooled OR= 1.47, 95% CI= 0.74–2.92	
		Currently unemployed		1 study (N=294) [10]	FS	LT	Largest significant OR= **2.37, 95% CI= 1.30–4.40**	
	Job satisfaction	Job insatisfaction	Kent PM, 2008 [26]	2 studies (N=414) [10, 15]	FS	LT	Pooled OR= **2.65, 95% CI= 1.27–5.50**	
				2 studies (N=480) [6, 19]	WP	LT	Pooled OR=0.82, 95% CI= 0.42-1.62	
	Driving time	Increased driving time	Kent PM, 2008 [26]	1 study (N= 854) [11]	WP	LT	Largest significant OR= **1.82, 95% CI= 1.03–3.22**	
	Job control	Less control of work	Kent PM, 2008 [26]	1 study (N= 854) [11]	WP	LT	Largest significant OR= **1.95, 95% CI= 1.02–3.72 a**	
	Work psychosocal factors	Increased psychosocial stress at work (hectic work, skill discretion…)	Steenstra IA, 2011 [28]*	2 studies (N=1,996) [12, 36]	WP	LT	OR= **2.16, 95%CI= 1.32–3.54**	OR=0.91, 95%CI= 0.81-1.01
	Work-related/derived injuries	Work-related/derived injuries (yes/no)	Steenstra IA, 2011 [28]*	2 studies (N=1,230) [5, 21]	WP	LT	OR= 1.54, 95%CI= 0.49–4.84	
	Alternative duties available	Modified duties not available	Steenstra IA, 2011 [28]*	2 studies (N=2,739) [11, 12]	WP	LT	OR ranged from **1.66, 95%CI= 1.22-2.46** to **1.91, 95%CI= 1.31–2.76**	
			Kent PM, 2008 [26]****	1 study (N=854) [11]	WP	LT	Largest significant OR= **1.99, 95% CI= 1.39–2.86**	
**Socio-occupational**	Claim related factors	Claim related factors (presence)(compensation, insurance, attorney involved)	Steenstra IA, 2011 [28]*	2 studies (N=2,001) [12, 18]	WP	LT	OR ranged from 0.66, 95%CI= 0.44–0.99 to 1.32, 95%CI=0.54-3.27	
**Social factors**	Social activity	Less social activity	Kent PM, 2008 [26]	2 studies (N=414) [10, 15]	FS	LT	Pooled OR= **2.31, 95% CI= 1.12–4.76**	
				1 study (N= 854) [11]	WP	LT	Largest significant OR= **2.79, 95% CI= 1.98–3.93**	

LBP= Low back pain; N= Sample size; [Ref] = References provided in Supplementary Table S5; OR= Odd ratio; NR= Not reported; LT: Long-term follow up (≥ 3 months); S/T: Sort-term follow-up (<3 months)

Outcome: P: Pain; FS: Functional status; WP: Work participation; R (c): Recovery c= slightly better" or "worse" score on two or more follow-up measurements

* Sample of individuals in acute phase of low back pain

**** Duplicate finding

Supplementary Table S9: Prognostic factors in Chronic LBP

Table S8. Prognostic factors for chronic LBP outcomes at long term (OR/Beta Coefficients)

Prognostic factor domain	Prognostic factor	Factor definition	Author, year [Ref]	Nº primary studies included (N) [Ref]	Outcome	Adjusted OR/Beta
**Chronic LBP (>3 meses)**						
**General health**	Physical activity	High self-reported physical activity	Oliveira CB, 2019 [40]	1 study (N=793) [52]	P	Unstandardized β= −0.2 (95% CI= −1.1 to −0.3), p=0.49
				2 studies (N=877) [53, 54]	FS	Unclear
**Physical impairment**	Abdominal muscle function	Worse Función de la musculatura abdominal (Basal dynamic morphometric measurements Transverse Abdomen)	Wong AY, 2013 [32]	1 studies (N=87) [55]	P	Unclear
**Psychological-cognitive**	Fear avoidance beliefs	High fear avoidance beliefs (FABQ y TSK)	Wertli MM, 2014 [35]	1 studies (N=681) [56]	P	OR= 1.20, 5%CI= 0.79–1.82
				1 studies (N=50) [31]	FS	Standardized β= 0.25, p= ns
				1 studies (N=822) [57]	WP	OR= **0.95, 95%CI= 0.91–0.99**
	Pain catastrophism	High pain catastrophism (PCS, CSQ, PRSS)	Wertli MM, 2014 [36]	3 studies (N=1,201) [24, 58, 59]	FS	Standardized β ranged from **0.35, p≤0.0001** to **0,11, p=0.001**

LBP= Low back pain; N= Sample size; [Ref] = References provided in Supplementary Table S5; OR= Odd ratio

Outcome: P: Pain; FS: Functional status; WP: Work participation

Bold results are statistically significant

Supplementary Table S10: Prognostic factors in Mixed-duration LBP

Table S10. Prognostic factors for mixed duration of LBP outcomes at long term (OR/Beta Coefficients)

Prognostic factor domain	Prognostic factor	Factor definition	Author, year [Ref]	Nº primary studies included (N) [Ref]	Outcome	Adjusted OR/Beta	Heterogeneity I^2 (p)	Crude OR/Beta	Heterogeneity I^2 (p)	Publication bias
**Acute to chronic LBP**										
**General health**	Physical activity	Higher self-reported physical activity	Oliveira CB, 2019 [40]	1 study (N=4,074) [60]	P	OR women= 0.97, 95% CI= 0.74-1.26, p= 0.83<br>OR men= 1.24, 95% CI= 0.93-1.67, p= 0.15	NA		NA	NA
				1 study (N=4,074) [60]	FS	OR women= 1.15, 95% CI= 0.9-1.47, p= 0.26<br>OR men= 1.19, 95% CI= 0.85-1.65), p= 0.31	NA		NA	NA
**Psychological-cognitive**	Fear avoidance beliefs	High fear avoidance beliefs (FABQ y TSK)	Wertli MM, 2014 [35]	1 study (N=165) [61]	WP	OR= **1.077, 95%CI= 1.05–1.11**	NA		NA	NA
				4 studies (N=2,449) [51, 62, 63]	FS	Ranged from OR= **0.91, 95%CI= 0.87–0.96** (for decrease RMQ≥ 30%) to Beta= **0.84, 95%CI= 0.25–1.44**).	NA		NA	NA
**Socio-occupational**	Work social support	Co-worker social support (low)	Campbell P, 2013 [30]	3 studies (ND) [36, 62, 64]	Rd	OR= **4.08, 95%CI= 1.59–10.05**	NA	Values ranged from Beta 0.2, p = 0.079 and OR 0.88, 95%CI= 0.64-1.21	NA	NA
		Supervisor social support (low)		2 studies (ND) [62, 64]	Rd	OR ranged from 1.07, 95%CI= 0.82-1.09 to 2.69, 95%CI= 0.85–8.44	NA		NA	NA
		General social support (low)		6 studies (ND) [35, 65-67, 68, 69]	Rd	Unclear	NA	OR= 1.16, 95%CI= 1.03-1.30	NA	NA
**Subacute/chronic LBP**										
**Psychological-cognitive**	Expectativas de recuperación	Positive recovery expectations (general expectations related to a future low back pain outcome)	Hayden JA, 2019 [34]	4 studies (N=1,820) [70-72a-b]	P*		NA	OR= 2.51, 95%CI= 0.81-7.82 (dichotomous measure) (only one study)	NA	NA
						Pooled OR= **1.15, 95%CI= 1.08-1.23** (continuous measure)(only two studies)	I^2 = 0% (p= 0,52)	Pooled OR= 1.13, 95%CI= 0.48-2.67 (continuous measure) (only two studies)	I^2 = 77% (p= 0.01)	NR
				8 studies (N=3,038) [70-76]	FS*	Pooled OR= 1.66, 95%CI= 0.66-4.22 (dichotomous measure) (only two studies)	I^2 = 0% (p= 0.72)	OR= **3.03, 95%CI= 1.14-8.08** (dichotomous measure) (only one study)	NA	NA
						Pooled OR= 1.40, 95%CI= 0.85-2.31 (continuous measure)	I^2 = 81% (p= 0)	Pooled OR= 1.56, 95%CI= 0.72-3.41 (continuous measure)	I^2 = 72% (p= 0.01)	NR
				15 studies (N=5,365) [12, 16, 38, 41, 45, 77-84]	WP*	Pooled OR= **2.43, 95%CI= 1.64-3.62** (dichotomous measure)	I^2 = 89% (p<0.0001)	Pooled OR= **4.11, 95%CI= 3.46-4.89** (dichotomous measure)	I^2 = 6% (p= 0.39)	Egger's test bias coefficiente= 2.79; p <0.001
						Pooled OR= 1.14, 95%CI= 0.95-1.37 (continuous measure) (only two studies)	I^2 = 54% (p= 0.14)	Pooled OR= 1.84, 95%CI= 0.53-6.37 (continuous measure)	I^2 = 90% (p= 0)	
				10 studies (N=7,766) [83, 85-93]	Re*	Pooled OR= **1.89, 95%CI= 1.49-2.41** (dichotomous measure)	I^2 = 0% (p= 0.78)	Pooled OR= **2.40, 95%CI= 1.32-4.37** (dichotomous measure)	I^2 = 49% (p= 0.14)	NR
						Pooled OR= **1.15, 95%CI= 1.07-1.24** (continuous measure)	I^2 = 81% (p= 0)	OR= **1.13, 95%CI= 1.11-1.15** (continuous measure) (only one study)	NA	NR
		Positive self-efficacy expectations (ability to execute behaviours to achieve a future outcome)	Hayden JA, 2019 [34]	1 study (N=107) [94]	P*	Beta= **9.44, SE= 3.94**	NA			
				2 studies (N=1,058) [70, 74]	FS*	Unclear	NA			
				1 study (N=135) [95]	Re*	OR= -0.34, p= 0.20	NA			
		Positive treatment expectations (future low back pain outcomes specifically related to ongoing treatment)	Hayden JA, 2019 [34]	2 studies (N=811) [76, 96]	FS*	Unclear	NA			

LBP= Low back pain; N= Sample size; [Ref] = References provided in Supplementary Table S5; OR= Odd ratio; NR= Not reported; NA= Not assessable

Outcome: P: Pain; FS: Functional status; WP: Work participation; R (d, e): Recovery d= recovery and/or return to work; e= important functional, pain or work participation recovery

NA: Not assessable
*Associated with good outcomes
Bold results are statistically significant
